# Variable importance measures for heterogeneous causal effects

**DOI:** 10.1093/biomtc/ujaf140

**Published:** 2025-12-24

**Authors:** Oliver J. Hines, Karla Diaz-Ordaz, Stijn Vansteelandt

**Affiliations:** 1 Department of Epidemiology, Columbia University, New York, NY, U.S.A; 2 Department of Statistical Science, University College London, U.K; 3 Department of Applied Mathematics, Computer Science and Statistics, Ghent University, Ghent, Belgium

**Keywords:** Causal inference, Conditional effects, Data-adaptive estimation, Effect modification

## Abstract

Motivated by applications in precision medicine and treatment effect heterogeneity, recent research has focused on estimating conditional average treatment effects (CATEs) using machine learning (ML). CATE estimates may represent complicated functions that provide little insight into the key drivers of heterogeneity. Therefore, we introduce nonparametric treatment effect variable importance measures (TE-VIMs), based on the mean-squared error (MSE) in predicting the individual treatment effect. More precisely, TE-VIMs represent the increase in MSE when variables are removed from the CATE conditioning set. We derive efficient TE-VIM estimators which can be used with any CATE estimation strategy and are amenable to ML estimation. We propose several strategies to calculate these VIMs (e.g. leave-one out, or keep-one in), using popular meta-learners for the CATE. We study the finite sample performance through a simulation study and illustrate their application using clinical trial data.

## 1 Introduction

In the medical and social sciences there is a longstanding interest in quantifying the heterogeneity in the effect of a treatment or intervention on a population. Understanding such heterogeneity is essential for informing scientific research and optimizing treatment decisions. Attention focused initially on subgroup analyses, which identify population subgroups that benefit most/least from treatment, to be evaluated further in potential future studies (Rothwell, 2005; Slamon et al., 2001). Typical challenges of subgroup analyses are selecting stratification variables in a systematic way and handling the resulting multiplicity problem. Endeavours to address these were soon followed by methodological developments on personalized medicine in causal inference, pioneered by Murphy (2003), with the primary focus being on policy learning; i.e., determining the treatment policy that minimizes some measure of population risk (van der Laan and Luedtke, 2014; Athey and Wager, 2021).

Recently, attention has partially shifted towards learning the conditional average treatment effect (CATE) *τ*(***x***) ≡ *E*(*Y* ^1^ − *Y* ^0^|***X*** = ***x***), where *Y^a^* is the outcome observed if treatment *A* were set to *a* ∈ {0, 1}, and ***X*** ∈ ℝ*^p^* are pre-treatment covariates (Athey and Imbens, 2016; Wager and Athey, 2018; Künzel et al., 2019; Kennedy, 2023). The CATE provides insight into the magnitude of the treatment effect for each individual and the optimal dynamic treatment rule (OTR), obtained from the sign of the CATE (VanderWeele et al., 2019).

These foregoing developments are important, but leave unanswered a key question: what are the key drivers of treatment effect heterogeneity? Answers of this question may inform about treatment mechanism, suggest future therapies, help compare clinical trial populations, or help quantify systematic treatment biases (e.g. due to race or socio-economic status). One pioneering proposal for CATE variable attribution is to extend random forest variable importance measures (VIMs) through the ‘causal forest’ estimator of the CATE (Athey et al., 2019; Wager and Athey, 2018). The resulting VIMs rely on the tree architecture of causal forests, and may inherently assign greater importance to continuous variables, or categorical variables with many categories (Grömping, 2009). VIMs based on specific modeling strategies (decisions trees, linear regression, etc.) are referred to as ‘algorithmic’ (Williamson and Feng, 2020). Whilst algorithmic-VIMs can provide useful insights, there remains a need for model-agnostic alternatives, especially when the chosen CATE estimating strategy does not have a well-established algorithmic-VIM, but also in order to compare VIMs following different CATE estimation procedures. Therefore, we propose treatment effect VIMs (TE-VIMs) that are nonparametric summary statistics, which measure the importance of variable subsets in predicting the individual treatment effect (ITE) *Y* ^1^ − *Y* ^0^. TE-VIMs are relatively easy to communicate to researchers already familiar with traditional goodness-of-fit methods such as ANOVA, and, unlike algorithmic-VIMs, allow researchers to compare heterogeneous treatment effect insights across different CATE learning algorithms.

More precisely, we consider the mean-squared-error *L*{*f*} ≡ *E*[{*Y* ^1^ − *Y* ^0^ − f (***X***)}^2^], which for arbitrary *f* : ℝ^*p*^ ↦ ℝ is not identified without strong assumptions on the joint distribution of (*Y*^1^, *Y*^0^) (Levy et al., 2021; Ding et al., 2016). One key insight is that *L*{*f*} = *E*[{*τ* (***X***) − *f* (***X***)}^2^]+ *E*{var(*Y* ^1^ − *Y* ^0^|***X***)} comprises a first term that is identified under standard causal assumptions and another that does not depend on *f*. Exploiting this decomposition, we define the TE-VIM Θ_*s*_ ≡ *L*{*τ*_*s*_}−*L*{*τ*}, where *τ*_*s*_(***x***) ≡ *E*(*Y* ^1^−*Y* ^0^|***X***_−*s*_ = ***x***_−*s*_) is the CATE conditional on ***X***_−*s*_, and ***u***_−*s*_ denotes the vector of all the components of ***u*** with index not in *s* ⊆ {1, …, *p*}. Note that *τ*_*s*_(***x***) only depends on ***x***_−*s*_, but we write it as a function of ***x*** to simplify notation. We interpret Θ_*s*_ = var{*τ* (***X***)} − var{*τ*_*s*_(***X***)} in terms of the variance var{*τ* (***X***)} of the treatment effect (VTE), a global measure of treatment effect heterogeneity due to Levy et al. (2021), see Supplement D.1. Specifically, Θ_*s*_ ≥ 0 represents the increase in CATE variance when variables in *s* are excluded from the conditioning set. Thus, Θ_*s*_ quantifies the treatment effect heterogeneity explained by ***X***_*s*_, beyond that already explained by ***X***_−*s*_, where ***u***_*s*_ denotes the vector of all components of ***u*** with index in *s*.

Moreover, TE-VIMs connect to regression-VIMs (Williamson et al., 2021; Zhang and Janson, 2020), also called ‘leave-out covariates’ (Lei et al., 2018; Verdinelli and Wasserman, 2024a), and the nonparametric VIM framework of Williamson et al. (2023). The latter framework covers VIMs that represent differences in value (negative risk) functions. Our work represents a step towards applying this framework in more complicated settings, such as in causal inference where identification may be a concern.

In Section 2 we derive efficient TE-VIM estimators and motivate the DR-learner of the CATE by interpreting our estimators in terms of pseudo-outcomes (Kennedy, 2023). Results on simulated data are presented in Section 3 and in Section 4 we use TE-VIMs to identify drivers of treatment effect heterogeneity in a clinical trial setting. In Section 5 we compare TE-VIMs with existing OTR-VIMs and outline extensions to continuous treatments.

## 2 Methodology

### 2.1 Motivating the estimand

We consider *n* i.i.d. observations (***z***_1_, …, ***z***_*n*_) of a random variable ***Z*** = (*Y, A*, ***X***) ~ *P*_0_ distributed according to an unknown distribution *P*_0_ ∈ ℳ and consisting of an ‘outcome’ *Y* ∈ ℝ, an ‘exposure’ or ‘treatment’ *A* ∈ {0, 1}, and covariates ***X*** ∈ ℝ^*p*^. In a slight abuse of notation, we let *p* denote the index set {1, …, *p*} so that *τ*_*p*_ is the average treatment effect (ATE) and Θ_*p*_ is the VTE, which we assume to be non-zero. Assuming consistency (*A* = *a* ⇒ *Y* = *Y* ^*a*^), conditional exchangeability (*Y*^*a*^ ⫫ *A*|***X*** for *a* = 0, 1), and positivity (0 < *π*(***X***) < 1 almost surely), the CATE is identified by *τ*(***x***) = *μ*(1, ***x***) − *μ*(0, ***x***), where *μ*(*a*, ***x***) ≡ *E*(*Y* |*A* = *a*, ***X*** = ***x***) and *π*(***x***) ≡ *E*(*A*|***X*** = ***x***) is the ‘propensity score’. We let ‖*f* (***Z***)‖ ≡ *E*[{*f* (***Z***)}^2^]^1/2^ denote the *L*_2_(*P*_0_) norm of an arbitrary function *f*, and when *f* is estimated from data, the expectation is taken only over the random inputs ***Z***. Assuming that ‖*τ*(***X***)‖ < ∞, then the TE-VIM Θ_*s*_ = ‖*τ*(***X***) − *τ*_s_(***X***)‖^2^ < ∞ is also finite. By construction, *s*′ ⊆ *s* ⊆ *p* implies ${\text{Θ}}_{s^{\prime }}\leq {\text{Θ}}_{s}\leq {\text{Θ}}_{p}$, i.e. the set *s*′ cannot be more important than *s*. Generally, the covariates used to define the CATE need not be the same as the covariates ***X*** required for conditional exchangeability to hold. E.g. one could consider the target estimand $L\left\{\tau_{s^{\prime }}\right\}-L\left\{\tau_{s}\right\}={\text{Θ}}_{s^{\prime }}-{\text{Θ}}_{s}$, which quantifies the importance of *s*′ with *s* treated as the full covariate set. This extension follows from results for Θ_*s*_, which we study in the current work.

The TE-VIM Θ_*s*_ is analogous to the regression-VIM *Ω*_*s*_ ≡ ‖*Y* − *μ*_*s*_(***X***)‖^2^ − ‖*Y* − *μ*(***X***)‖^2^ due to Williamson et al. (2021), which replaces the ITE *Y* ^1^ − *Y*^0^ with *Y*, and hence *τ* (***x***) with *μ*(***x***) ≡ *E*(*Y* |***X*** = ***x***) and *τ*_*s*_(***x***) with *μ*_*s*_(***x***) ≡ *E*(*Y* |***X***_−*s*_ = ***x***_−*s*_). The two proposals differ, however, in how the VIM is scaled with Williamson et al. (2021) defining the scaled regression-VIM Ω_*s*_*/*var(*Y*) in analogy with the familiar *R*^2^ statistic. Since var(*Y* ^1^ − *Y*^0^) is not easily identified, we instead propose scaled TE-VIMs as Ψ_*s*_ ≡ Θ_*s*_*/*Θ_*p*_ = 1−var{*τ*_*s*_(***X***)}*/*Θ_*p*_. Like an *R*^2^ statistic, we interpret Ψ_*s*_ ∈ [0, 1] as the proportion of treatment effect heterogeneity explained by ***X***_−*s*_ compared with ***X***. For instance, under the linear model *E*(*Y^a^*|***X*** = ***x***) = *μ*(*a*, ***x***) = *β*(***x***) + *aτ* (***x***), where *β*(***x***) and *τ* (***x***) are both linear in ***x***, Ψ_*s*_ is the limiting *R*^2^ value obtained from a linear regression of the effect modifier *τ* (***X***) on ***X***_−*s*_. Moreover, Ψ_*s*_ and Ω_*s*_*/*var(*Y*) are both invariant to linear transformations of the outcome and invertible component-wise transformations of ***X***, see Supplement D.2 for details. In practice, VIM scaling makes little difference when using Ψ_*s*_ and ${\text{Ψ}}_{s^{\prime }}$ to compare the relative importance of *s* and *s*′. Instead, the main decision for investigators is which covariate sets should be compared, and we identify the following modes of operation in this regard:

1. **Leave-one-out (LOO)**: The set *s* contains a single covariate of interest. This mode may ‘under represent’ importance when covariates are highly correlated.
2. **Keep-one-in (KOI)**: The set *s* contains all but a single covariate of interest. This mode may ‘over represent’ importance when covariates are highly correlated, and is less sensitive to multi-covariate interactions.
3. **Shapley values**: TE-VIMs for all possible (2^*p*^) covariate combinations are considered and aggregated in a game theoretic manner (Owen and Prieur, 2017; Williamson and Feng, 2020). This is a theoretically appealing compromise between LOO and KOI, but may be computationally impractical for even modest *p* and render clinical interpretation more subtle. A definition of TE-VIM Shapley values is given in Supplement D.3.
4. **Covariate grouping**: Domain specific knowledge is used to group covariates, simplifying the above modes by considering covariates block-wise (e.g. comparing biological vs. non-biological factors).

Note that we use ‘under’ and ‘over’ represent in a relative sense, since the ground truth variable importance depends on the importance definition used. See e.g. Hama et al. (2022); Verdinelli and Wasserman (2024b) for recent discussions on this topic and Tang and Westling (2025) for variable selection proposals. In Section 4 we demonstrate the LOO and KOI modes through an applied example.

### 2.2 CATE estimation

Estimation of TE-VIMs will rely on initial CATE estimates, obtained via flexible machine learning based methods, which we review first. CATE estimation is challenging since common machine learning algorithms (random forests, neural networks, boosting etc.) are designed for mean outcome regression, e.g. by minimizing the mean squared error loss. CATE estimation strategies therefore either modify existing machine learning methods to target CATEs, e.g. Athey et al. (2019) modify the random forest algorithm for CATE estimation. Alternatively, ‘metalearning’ strategies decompose CATE estimation into a sequence of sub-regression problems, which can be solved using off-the-shelf machine learning algorithms (Künzel et al., 2019; Kennedy, 2023).

In the current work we focus on two metalearning algorithms which, following the naming convention of Künzel et al. (2019) and Kennedy (2023), we refer to as the T-learner and the DR-learner. The T-learner is based on the decomposition *τ* (***x***) = *μ*(1, ***x***) − *μ*(0, ***x***), and estimates the CATE by ${\hat{\tau }}^{\left(T\right)}(x)\equiv \hat{\mu }(1,x)-\hat{\mu }(0,x),$ where $\hat{\mu }(a,x)$ represents an estimate of *μ*(*a*, ***x***) obtained by a regression of *Y* on *X* using observations where *A* = *a*. The T-learner, however is problematic for two main reasons. Firstly, whilst regularization methods can be used to control the smoothness of $\hat{\mu }(a,x)$, the same is not true of ${\hat{\tau }}^{\left(T\right)}(x)$ which may be erratic. Slow convergence rates affecting $\hat{\mu }(a,x)$ may therefore propagate into ${\hat{\tau }}^{\left(T\right)}(x)$. Secondly, $\hat{\mu }(1,x)$ is chosen to make an optimal bias-variance trade-off over the covariate distribution of the treated population. Likewise, $\hat{\mu }(0,x)$ is chosen to make an optimal bias-variance trade-off over the covariate distribution of the untreated population. When there is poor overlap between the treated and untreated subgroups, then ${\hat{\tau }}^{\left(T\right)}(x)$ may fail to deliver an optimal bias-variance trade-off over the population covariate distribution, making the T-learner potentially poorly targeted towards CATE estimation.

The DR-learner (Kennedy, 2023; Luedtke and van der Laan, 2016; van der Laan, 2013) is an alternative metalearning algorithm based on the decomposition *τ* (***x***) = *E*{*φ*(***Z***)|***X*** = ***x***} where, for ***z*** = (*y*, *a*, ***x***)

(1) $$\varphi (z)\equiv \{y-\mu (a,x)\}\frac{a-\pi (x)}{\pi (x)\{1-\pi (x)\}}+\mu (1,x)-\mu (0,x).$$

is called the ‘pseudo outcome’, or the augmented inverse propensity weighted score (Robins et al., 1994), and acts like the ITE in expectation. The DR-learner first estimates *μ*(*a*, ***x***) and *π*(***x***) to obtain the pseudo-outcome estimator $\hat{\varphi }(Z)$, where *μ*(*a*, ***x***) and *π*(***x***) in (1) are replaced with estimates $\hat{\mu }(a,x)$ and $\hat{\pi }(x)$.

In a second step, the estimated pseudo-outcome, $\hat{\varphi }(Z)$ is regressed on covariates ***X*** to obtain ${\hat{\tau }}^{\left(DR\right)}(x)$. A sample splitting scheme is also recommended, whereby the regression steps to obtain $\hat{\mu }(a,x),\hat{\pi }(x)$, and ${\hat{\tau }}^{\left(DR\right)}(x)$ are performed on three independent samples.

The DR-learner alleviates the issues related to the T-learner since the complexity of ${\hat{\tau }}^{\left(DR\right)}(x)$ can be controlled by regularizing the regression in the final stage of the procedure, mitigating concerns regarding the smoothness of the T-learner. With regard to consistency, taking expectation over the random inputs ***Z***, the square of $E\{\hat{\varphi }(Z)|X=x\}-\tau (x)$ is bounded above by the product of the squared estimation errors of the propensity score and regression estimators (up to constant scaling). In practice, this means that the regression of $\hat{\varphi }(Z)$ on ***X*** mimics the oracle regression of *φ*(***Z***) on ***X*** provided that

$$(\text{A}1)\;\|\{\pi (X)-\hat{\pi }(X)\}\{\mu (a,X)-\hat{\mu }(a,X)\}\|=o_{P}\left(n^{-1/2}\right)\;\text{for}\;a=0,1.\;$$

and a suitable cross-fitting procedure is used. (A1) implies that one can trade-off accuracy in the outcome and propensity score estimators, a property known as double robustness, hence the name ‘DR-learner’. Note that the *n*^−1*/*2^ rate in (A1) differs from the ‘stability’ condition provided for the DR-learner by Kennedy (2023, Proposition 1), as it reflects what will be required by our TE-VIM estimators in Section 2.3.

Estimation of the CATE *τ*_*s*_(***x***) is complicated by the fact that one cannot assume that *Y* ⫫ *A*|***X***_−*s*_ for an arbitrary subset of covariates *s*, a problem that is sometimes referred to as ‘runtime confounding’ (Coston et al., 2020). The DR-learner readily accommodates runtime confounding through the identity *τ*_*s*_(***x***) = *E*{*φ*(***Z***)|***X***_−*s*_ = ***x***_−*s*_}. This identity implies that one may estimate *τ*_*s*_(***x***) by regressing $\hat{\varphi }(Z)$ on ***X***_−*s*_, i.e. modifying the final regression step of the DR-learner.

We recommend a metalearner for *τ*_*s*_(***x***) based on the identity *τ*_*s*_(***x***) = *E*{*τ* (***X***)|***X***_−*s*_ = ***x***_−*s*_}. Specifically, we propose estimating *τ*_*s*_(***x***) by regressing initial CATE estimates, $\hat{\tau }(X)$ on ***X***_−*s*_ via a machine learning algorithm. Like the DR-learner, one can regularize the resulting CATE estimator ${\hat{\tau }}_{s}(x)$. We advocate this approach, since it usually results in estimates of *τ*_*s*_(***x***) which are compatible with those of *τ* (***x***), in the sense of respecting the fact that the two conditional means are related. For instance, we expect that *E*{*φ*(***Z***)} = *E*{*τ* (***X***)} = *E*{*τ*_*s*_(***X***)}, but these equalities are generally violated for the corresponding estimated quantities $E\{\hat{\varphi }(Z)\},E\{\hat{\tau }(X)\},$ and $E\left\{{\hat{\tau }}_{s}(X)\right\}$.

### 2.3 TE-VIM estimation

#### 2.3.1 Estimation of Θ_*s*_

We consider estimators based on the efficient influence curve (IC) of Θ_*s*_ under the non-parametric model. Briefly, ICs are mean zero functions that characterize the sensitivity of so-called pathwise differentiable estimands to small changes in the data generating law. Thus, ICs are useful for constructing efficient estimators and determining their asymptotic distribution, see e.g. Hines et al. (2022) for an introduction.

TE-VIMs fall under the VIM framework of Williamson et al. (2023), for which generic IC results are available. These results cannot be directly applied, however, since the risk *L*{*f*} is not identified. Instead, we consider that Θ_*s*_ = *L*_*U*_ {*τ*_*s*_} − *L*_*U*_ {*τ*} where

$$L_{U}\{f\}=E\left[{\left\{U+\tau (X)-f(X)\right\}}^{2}\right]=E\left[{\left\{\tau (X)-f(X)\right\}}^{2}\right]+E\left(U^{2}\right)$$

and *U* is a random variable such that *E*(*U*|***X***) = 0. Note that *U* = *Y*^1^ −*Y*^0^ −*τ*(***X***) recovers the unidentified risk *L*{*f*} and *U* = *φ*(***Z***) − *τ* (***X***) recovers the DR-learner population risk. To simplify derivations we consider the risk L*{*f*} obtained by setting *U* = 0. Theorem 3 of Williamson et al. (2023) states that, for this risk, there is no price to pay to estimate its minimizer *τ*_*s*_(***x***), insofar as the IC for L*{*τ*_*s*_} that is derived when *τ*_*s*_(***x***) is known is the same as that derived when *τ*_*s*_(***x***) is unknown. In Supplement A we use point mass contamination to show that, for known *f*, the IC of L*{*f*} at a single observation ***z*** is {*φ*(***z***) − *f*(***x***)}^2^ − {*φ*(***z***) − *τ* (***x***)}^2^ − L* {*f*}. Hence, applying the aforementioned Theorem, the IC of Θ_*s*_ is

(2) $$\phi_{s}(z)\equiv {\left\{\varphi (z)-\tau_{s}(x)\right\}}^{2}-{\left\{\varphi (z)-\tau (x)\right\}}^{2}-{\text{Θ}}_{s}.$$

The interpretation of *φ*(***Z***) as a pseudo-outcome, which plays the role of the unobserved ITE *Y*^1^ − *Y*^0^, holds in the present context. To see why, we compare (2) to the IC of Ω_*s*_ by Williamson et al. (2021), {*y* − *μ*_*s*_(***x***)}^2^ − {*y* − *μ*(***x***)}^2^ − Ω_s_, which is of the same form as (2), but with the outcome y replacing the pseudo-outcome *φ*(***z***).

The IC in (2) may be used to construct efficient estimating equation estimators of Θ_*s*_ by setting (an estimate of) the sample mean IC to zero. This strategy is equivalent to the so-called one-step correction outlined in Supplement B. We thus obtain the estimator

(3) $${\hat{\Theta }}_{s}\equiv n^{-1}\sum_{i=1}^{n}\left[{\{\hat{\varphi }\left(z_{i}\right)-{\hat{\tau }}_{s}\left(x_{i}\right)\}}^{2}-{\{\hat{\varphi }\left(z_{i}\right)-\hat{\tau }\left(x_{i}\right)\}}^{2}\right],$$

where superscript hat denotes consistent estimators. In practice, we recommend a cross-fitting procedure of the type described in Algorithms SS-A and SS-B, to obtain the fitted models and evaluate the estimators using a single sample (Chernozhukov et al., 2018; Zheng and van der Laan, 2011). We discuss the reasons for sample splitting with reference to Theorem 1, which gives conditions under which ${\hat{\Theta }}_{s}$ is regular asymptotically linear (RAL).

##### Theorem 1

Assume that there exist constants *ϵ*, *K*, *δ* ∈ (0, ∞) such that almost surely $\hat{\pi }(X)\in (\epsilon ,1-\epsilon ),\mathrm{var}\{\varphi (Z)|X\}<K$, and $∥\hat{\tau }(X)-{\hat{\tau }}_{s}(X)∥<\delta .$ Suppose also that at least one of the following two conditions hold:

1. **Sample splitting**: $\hat{\pi }(x),\hat{\mu }(x),\hat{\tau }(x)$, and ${\hat{\tau }}_{s}(x)$ are obtained from a sample indepen-dent of the one used to construct ${\hat{\Theta }}_{s}$.
2. **Donsker condition**: The quantities ${\left\{\varphi (Z)-\hat{\tau }(X)\right\}}^{2},{\left\{\varphi (Z)-{\hat{\tau }}_{s}(X)\right\}}^{2}$, and $\left\{\hat{\tau }(X)-{\hat{\tau }}_{s}(X)\}\hat{\varphi }(Z\right)$ fall within a *P*_0_-Donsker class with probability approaching 1.

Finally assume (A1) holds, and (A2) that $\|\tau (X)-\hat{\tau }(X)\|\;$ and $\left||\tau_{s}(X)-{\hat{\tau }}_{s}(X)|\right|$ are both *o*_*P*_ (*n*^−1*/*4^). Then ${\hat{\Theta }}_{s}$ is asymptotically linear with IC *ϕ*_*s*_(***Z***), and hence ${\hat{\Theta }}_{s}$ converges to Θ_*s*_ in probability, and for Θ_*s*_ > 0 then $n^{1/2}\left({\hat{\Theta }}_{s}-{\text{Θ}}_{s}\right)$ converges in distribution to a mean-zero normal random variable with variance ‖*Φ*_*s*_(***Z***)‖^2^.

Assumptions (A1-A2) both require nuisance function estimators to converge at sufficiently fast rates. The requirement for *n*^1*/*4^ rate convergence in (A2) is standard in the recent VIM framework of Williamson et al. (2023), whilst (A1) is additionally required to control for errors in estimating the pseudo-outcomes.

Together, (A1-A2) suggest that the DR-learner may be preferred over the T-learner due to its robustness. In particular, the T-learner of the CATE satisfies $∥\tau (X)-{\hat{\tau }}^{\left(T\right)}(X)∥=o_{P}\left(n^{-1/4}\right)$, provided that $\|\mu (a,X)-\hat{\mu }(a,X)\|=o_{P}\left(n^{-\alpha }\right)$, with *α* ≥ 1/4 for *a* = 0, 1. (A1) then implies that $\left\|\pi (X)-\hat{\pi }(X)\right\|$ must be at least *o*_*P*_ (*n*^−1*/*2+*α*^). I.e. the propensity score estimator may converge at a slower rate, if the outcome estimator converges at a faster rate, but the converse is not true since fast convergence of the outcome estimator is needed to assure sufficiently fast convergence of the T-learner. This is unsatisfying for example in clinical trial settings, where the exposure is randomized and propensity scores are known.

The DR-learner, however, satisfies $∥\tau (X)-{\hat{\tau }}^{\left(DR\right)}(X)∥=o_{P}\left(n^{-1/4}\right)$ when (A1) holds and $∥E\{\hat{\varphi }(Z)|X\}-{\hat{\tau }}^{\left(DR\right)}(X)∥=o_{P}\left(n^{-1/4}\right)$), i.e. when the final DR-learning regression estimator is consistent at *n*^−1/4^ rate. Applying the same reasoning as before, (A1) implies that $\left\|\mu (A,X)-\hat{\mu }(A,X)\right\|$ can be *o*_*P*_ (*n*^−*α*^), if $\left\|\pi (X)-\hat{\pi }(X)\right\|$ is *o*_*P*_ (*n*^−1*/*2+*α*^), for any *α* ∈ (0, 1/2). In other words, the outcome estimator is allowed to converge at a slower rate, provided the propensity score estimator converges at a faster rate and vice-versa, which marks an improvement over the T-learner, at the expense of an additional requirement on the final DR-learning step. The requirement on the DR-learning step, however, will likely be weaker than that on outcome estimator, since *τ* (***x***) is likely smoother than *μ*(*a*, ***x***), e.g. when the CATE depends only on a subset of ***X***.

The Donsker condition in Theorem 1 controls the ‘empirical process’ term in the estimator expansion (Newey and Robins, 2018; Hines et al., 2022). This condition is usually not guaranteed to hold when flexible machine learning methods are used to estimate nuisance functions. Fortunately, cross-fitting using two or more splits of the data offers a way of avoiding Donsker conditions, at the expense of making nuisance functions more computationally expensive to learn (Chernozhukov et al., 2018; Zheng and van der Laan, 2011).

#### 2.3.2 Importance testing

One property shared by ${\hat{\Theta }}_{s}$ and the analogous Ω_*s*_ estimator (Williamson et al., 2021) concerns their behavior under the zero-importance null hypothesis, *H*_0_ : Θ_*s*_ = 0. For TE-VIMs, *H*_0_ corresponds to treatment effect homogeneity *τ* (***x***) = *τ*_*s*_(***x***), in which case *ϕ*_*s*_(***Z***) = 0. This IC degeneracy makes *H*_0_ difficult to test, since ${\hat{\Theta }}_{s}$ is not necessarily asymptotically normal. For this reason, Theorem 1 considers the asymptotic distribution only when Θ_*s*_ > 0. One solution to the IC degeneracy problem is to estimate var{*τ* (***X***)} and var{*τ*_*s*_(***X***)}^2^ using efficient estimators in separate samples (Williamson et al., 2023). Each estimand has a non-zero IC provided that var{*τ*_*s*_(***X***)} > 0, despite both ICs being identical under *H*_0_. Thus both estimators are independent and asymptotically normal, hence their difference (an estimator of Θ_*s*_) is also asymptotically normal even when Θ_*s*_ = 0. One therefore obtains a valid Wald-type test for *H*_0_, at the expense of using an estimator for Θ_*s*_, which is inefficient because the component estimators are estimated using only half of the data. Similarly, one could test the zero-VTE null hypothesis (var{*τ* (***X***)} = 0) by estimating *E*{*τ*^2^(***X***)} and *E*{*τ* (***X***)}_2_ using efficient estimators in separate samples and taking their difference. Such approaches are an active area of research and evaluating them in the context of TE-VIMs is beyond the scope of the current work (Hudson, 2023; Guo and Shah, 2025). Moreover, the distribution of ${\hat{\Theta }}_{s}$ under *H*_0_ may depend on higher-order pathwise derivatives of the estimand, which are also an open research area (Carone et al., 2018).

#### 2.3.3 Estimation of Ψ_*s*_

The scaled TE-VIM Ψ_*s*_ ≡ Θ_*s*_/Θ_*p*_, has IC, Φ_*s*_(***z***) ≡ {*ϕ*_*s*_(***z***) − Ψ_*s*_*ϕ*_*p*_(***z***)}/Θ_*p*_, where *ϕ*_*p*_(***z***) denotes (2) for the index set *s* = *p*. This IC implies an estimating equations estimator, ${\hat{\Psi }}_{s}={\hat{\Theta }}_{s}/{\hat{\Theta }}_{p}$, where ${\hat{\Theta }}_{p}$ is the VTE estimator obtained when ${\hat{\tau }}_{s}(x)$ in (3) is replaced with an ATE estimate ${\hat{\tau }}_{p}$.

The estimators ${\hat{\Theta }}_{p}$ and ${\hat{\Psi }}_{s}$ both rely on an ATE estimator ${\hat{\tau }}_{p}$. The IC of the ATE, *φ*(***z***) − *τ*_*p*_, implies an efficient estimator ${\hat{\tau }}_{p}^{*}\equiv n^{-1}\sum_{i=1}^{n}\hat{\varphi }\left(z_{i}\right)$, known as the augmented inverse propensity weighted (AIPW) estimator (Robins et al., 1994). We recommend the AIPW estimator in the current context since ${\hat{\Theta }}_{p}$ and ${\hat{\Psi }}_{s}$ are locally insensitive to small perturbations in ${\hat{\tau }}_{p}$ about ${\hat{\tau }}_{p}^{*}$. To see why, consider the partial derivative

$$\frac{\partial {\hat{\Theta }}_{p}}{\partial {\hat{\tau }}_{p}}=\frac{\partial }{\partial {\hat{\tau }}_{p}}n^{-1}\sum_{i=1}^{n}\left[{\left\{\hat{\varphi }\left(z_{i}\right)-{\hat{\tau }}_{p}\right\}}^{2}-{\{\hat{\varphi }\left(z_{i}\right)-\hat{\tau }\left(x_{i}\right)\}}^{2}\right]=-2\left({\hat{\tau }}_{p}^{*}-{\hat{\tau }}_{p}\right),$$

which is zero at ${\hat{\tau }}_{p}={\hat{\tau }}_{p}^{*}$. This orthogonality means that uncertainty in the AIPW estimator can be ignored when estimating Θ_*p*_, see Vermeulen and Vansteelandt (2015) for details.

Like *ϕ*_*s*_(***z***), Ф_*s*_(***z***) degenerates to Ф_*s*_(***z***) = 0 when Ψ_*s*_ = 0 or when Ψ_*s*_ = 1, i.e. Θ_*s*_ = 0 or Θ_*s*_ = Θ_*p*_. For this reason, the asymptotic normality of ${\hat{\Psi }}_{s}$, described in Theorem 2, holds only for Ψ_*s*_ ∈ (0, 1), i.e. when covariates in s account for some, but not all, heterogeneity. Similar estimators for log(Θ_*s*_) and logit(Ψ_*s*_) are derived in Supplement D.5, which may be used to construct alternative bounded estimators ${\hat{\Theta }}_{s}^{*}>0$ and ${\hat{\Psi }}_{s}^{*}\in (0,1)$.

##### Theorem 2

Assume that the conditions in Theorem 1 are satisfied, Θ_*p*_ > 0, and there exists *δ* ∈ (0, ∞) such that $||\hat{\tau }(X)-{\hat{\tau }}_{p}^{*}||<\delta$. Then ${\hat{\Psi }}_{s}$, with the ATE estimated by ${\hat{\tau }}_{p}={\hat{\tau }}_{p}^{*}$, is asymptotically linear with IC, Ф_*s*_(***Z***), and hence ${\hat{\Psi }}_{s}$ converges to Ψ_*s*_ in probability, and for Ψ_*s*_ ∈ (0, 1) then $n^{1/2}\left({\hat{\Psi }}_{s}-{\text{Ψ}}_{s}\right)$ converges in distribution to a mean-zero normal random variable with variance ‖Φ_*s*_(***Z***)‖^2^.

#### 2.3.4 Plug-in estimation

A common framework for constructing debiased estimators is through targeted maximum likelihood estimators (TMLEs) (van der Laan and Gruber, 2016). TMLEs are ‘plug-in’, in that they are defined through estimand mappings, e.g. Θ_*s*_ : *ℳ* ↦ [0, ∞) evaluated at a distributoin estimate ${\hat{P}}_{n}\in ℳ$. Despite the apparent similarity of ${\hat{\Theta }}_{s}$ with the representation Θ_*s*_ = *E* [{*φ*(***Z***) − *τ*_*s*_(***X***)}^2^ − {*φ*(***Z***) − *τ* (***X***)}^2^], the estimators ${\hat{\Theta }}_{s},{\hat{\Theta }}_{p},{\hat{\Psi }}_{s}$ are not plug-in. This is evident from the fact that ${\hat{\Theta }}_{s}$ may take negative values, but such codomain violations are not possible for plug-in estimators. We emphasize this point since Williamson et al.(2021) use ‘plug-in’ to refer to representation similarities, and their Ω_*s*_ estimator is also not plug-in in the estimand mapping sense. Moreover, TMLEs for Θ_*s*_ are challenging, since the targeting step must target compatible estimators for *μ*(***x***_*i*_) and *μ*_*s*_(***x***_*i*_) simultaneously. The VTE does not suffer this issue, with TMLEs proposed by Levy et al. (2021). TMLEs for TE-VIMs are investigated by Li et al. (2023).

#### 2.3.5 Algorithms

The estimators ${\hat{\Theta }}_{s},{\hat{\Theta }}_{p}$, and ${\hat{\Psi }}_{s}$ are indexed by the choice of pseudo-outcome and CATE estimators. Generally, we are unrestricted in the choice of CATE metalearner, and outcome and propensity score learners. We propose two Algorithms based on the T- and DR-learners, with and without sample splitting. In Algorithm noSS substeps marked (A) and (B) refer to the T- and DR-learners respectively. Where the algorithms require models to be ‘fitted’, any suitable regression method/learner can be used.

Both algorithms return pseudo-outcome and CATE estimates, ${\left\{{\hat{\varphi }}_{i}\right\}}_{i=1}^{n},{\left\{{\hat{\tau }}_{i}\right\}}_{i=1}^{n}$,, and ${\left\{{\hat{\tau }}_{s,i}\right\}}_{i=1}^{n}$, which can be used to obtain ${\hat{\tau }}_{p}=n^{-1}\sum_{i=1}^{n}{\hat{\varphi }}_{i}$ and the uncentered $\text{ICs}{\hat{\phi }}_{i,s}={\left\{{\hat{\varphi }}_{i}-{\hat{\tau }}_{s,i}\right\}}^{2}-{\left\{{\hat{\varphi }}_{i}-{\hat{\tau }}_{i}\right\}}^{2}\;$ and ${\hat{\phi }}_{i,p}={\left\{{\hat{\varphi }}_{i}-{\hat{\tau }}_{p}\right\}}^{2}-{\left\{{\hat{\varphi }}_{i}-{\hat{\tau }}_{i}\right\}}^{2}$ which imply the estimators ${\hat{\Theta }}_{s}=n^{-1}\sum_{i=1}^{n}{\hat{\phi }}_{i,s},{\hat{\Theta }}_{p}=n^{-1}\sum_{i=1}^{n}{\hat{\phi }}_{i,p}$ and ${\hat{\Psi }}_{s}={\hat{\Theta }}_{s}/{\hat{\Theta }}_{p}$, with variances respectively estimated by $n^{-2}\sum_{i=1}^{n}{\left({\hat{\phi }}_{i,s}-{\hat{\Theta }}_{s}\right)}^{2},n^{-2}\sum_{i=1}^{n}{\left({\hat{\phi }}_{i,p}-{\hat{\Theta }}_{p}\right)}^{2}$ and ${\left(n{\hat{\Theta }}_{p}\right)}^{-2}\sum_{i=1}^{n}{\left({\hat{\phi }}_{i,s}-{\hat{\Psi }}_{s}{\hat{\phi }}_{i,p}\right)}^{2}$. The algorithms differ in their CATE metalearner and use of sample splitting. Comparing Algorithms SS-A and SS-B, the DR-learner requires additional cross-fitting because it is trained on pseudo-outcomes estimates that are learned from a separate sample. As such, Algorithm SS-B has *𝒪*(*K*^2^) regression operations compared with *𝒪*(*K*) for SS-A.

### Algorithm noSS: No sample splitting

(1) Fit $\hat{\mu }(.,.)$ and $\hat{\pi }(.)$. Use these fitted models to obtain ${\hat{\varphi }}_{i}\equiv \hat{\varphi }\left(z_{i}\right)$.
(2) (A) Use the model for $\hat{\mu }(.,.)$ from Step 1, to obtain $\hat{\tau }(x)\equiv \hat{\mu }(1,x)-\hat{\mu }(0,x)$. Or (B) Fit $\hat{\tau }(.)$ by regressing $\hat{\varphi }(Z)$ on ***X***. After doing (A) or (B), use the fitted models to obtain ${\hat{\tau }}_{i}\equiv \hat{\tau }\left(x_{i}\right)$.
(3) Fit ${\hat{\tau }}_{s}(.)\;$ by regressing $\hat{\tau }(X)$ on ***X***_−*s*_. Use the fitted model to obtain ${\hat{\tau }}_{s,i}\equiv {\hat{\tau }}_{s}\left(x_{i}\right)$.
(4) Optionally repeat Step 3 for other covariate sets of interest.

### Algorithm SS-A: Sample splitting with the T-Learner

(1) Split the data into *K* ≥ 2 folds. **For** each fold *k*: Fit $\hat{\mu }(.,.)$ and $\hat{\pi }(.)$ using the data set excluding fold *k*. Use these fitted models to obtain${\hat{\varphi }}_{i}\equiv \hat{\varphi }\left(z_{i}\right)$ and ${\hat{\tau }}_{i}\equiv \hat{\mu }(1,x_{i})-\hat{\mu }(0,x_{i})$ for *i* in fold *k*.
(2) Fit ${\hat{\tau }}_{s}(.)\;$ by regressing $\hat{\mu }(1,X)-\hat{\mu }(0,X)$ on ***X***_−*s*_ using the data excluding fold *k*. Use the fitted model to obtain ${\hat{\tau }}_{s,i}\equiv {\hat{\tau }}_{s}\left(x_{i}\right)$ for *i* in fold *k*.
(3) Optionally repeat Step 2 for other covariate sets of interest. **End for**.

### Algorithm SS-B: Sample splitting with the DR-Learner

(1) Split the data into *K* ≥ 3 folds. **For** each pair of folds *j* ≠ *k*: Fit $\hat{\mu }(.,.)$ and $\hat{\pi }(.)$ using the data set that excludes both folds *j* and *k*. Use these fitted models to obtain ${\hat{\varphi }}_{i}^{\left(k\right)}\equiv \hat{\varphi }\left(z_{i}\right)$ for *i* in fold *j*, and ${\hat{\varphi }}_{i}^{\left(j\right)}\equiv \hat{\varphi }\left(z_{i}\right)$ for *i* in fold *k*. **End for**.
(2) **For** each fold *k*: Fit $\hat{\tau }(.)$ by regressing ${\hat{\varphi }}^{\left(k\right)}(Z)$ on ***X*** using the data excluding fold *k*. Use the fitted models to obtain ${\hat{\tau }}_{i}\equiv \hat{\tau }\left(x_{i}\right)$ for *i* in fold *k*.
(3) Obtain ${\hat{\varphi }}_{i}\equiv {\left(K-1\right)}^{-1}\Sigma_{j\neq k}{\hat{\varphi }}^{\left(j\right)}\left(z_{i}\right)$ for *i* in fold *k*.
(4) Fit ${\hat{\tau }}_{s}(.)$ by regressing $\hat{\tau }(X)$ on ***X***_−*s*_ using the data excluding fold *k*. Use the fitted model to obtain ${\hat{\tau }}_{s,i}\equiv {\hat{\tau }}_{s}\left(x_{i}\right)$ for *i* in fold *k*.
(5) Optionally repeat Step 4 for other covariate sets of interest. **End for**.

## 3 Simulation study

We compared all Algorithms (*K* = 8 folds) under three data generating processes (DGPs). For each, generalized additive models (GAMs) were used to estimate *μ*(*a*, ***x***), *π*(***x***), *τ*_*s*_(***x***), and in the case of the DR-learner, *τ*(***x***). GAMs are flexible spline smoothing models implemented in the mgcv R package (Wood et al., 2016), and for DGPs 1 and 2, (*X*_1_, *X*_2_) interaction terms were included. Propensity score models were additive on the logit scale.

### DGP 1

We generated 500 datasets for each size *n* ∈ {500, 1000, 2000, 3000, 4000, 5000} according to *X*_1_, *X*_2_ ~ Uniform(−1, 1), *A* ~ Bernoulli{expit(−0.4*X*_1_+0.1*X*_1_*X*_2_)}, $\tau^{\left(1\right)}(X)=X_{1}^{3}+1.4X_{1}^{2}+25X_{2}^{2}/9$, and $Y\sim N_{1}\left(X_{1}X_{2}+2X_{2}^{2}-X_{1}+A\tau^{\left(1\right)}(X),1\right)$ where 𝒩_*p*_(0, Σ) denotes a *p*-dimensional normal variable with mean *μ* and covariance matrix Σ. In this case *τ*_*p*_ = 1.39 and Θ_*p*_ = 1.003. Since we consider only two covariates, the LOO and KOI TE-VIM modes are equivalent, with Θ_1_ = 0.32, Θ_2_ = 0.69, Ψ_1_ = 0.32, and Ψ_2_ = 0.68.

### DGP 2

The setup in DGP 1, but with *τ*^(1)^(***X***) replaced with *τ*^(2)^(***X***) = *τ*^(1)^(***X***)/10. In this way, the relative importance of *X*_1_, *X*_2_ are unchanged, but the overall effect size and heterogeneity is much smaller. This results in *τ*_*p*_ = 0.139, Θ_*p*_ = 0.01003, Θ_1_ = 0.0032 and Θ_2_ = 0.0069, but the scaled TE-VIMs Ψ_1_ = 0.32 and Ψ_2_ = 0.68 are the same as DGP 1.

### DGP 3

We generated 500 datasets with *n* = 5000 according to

$$\left(X_{1},X_{2}\right),\left(X_{3},X_{4}\right),\left(X_{5},X_{6}\right)\sim N_{2}\left(0,\left(\begin{array}{cc} 1 & 0.5\\ 0.5 & 1 \end{array}\right)\right),$$

*A* ~ Bernoulli{expit(−0.4*X*_1_ +0.1*X*_1_*X*_2_+0.2*X*_5_)}, *τ*^(3)^(***X***) = X_1_+2*X*_2_+*X*_3_, *Y* ~ 𝒩_1_(*X*_3_ − *X*_6_ + *Aτ*^(3)^(***X***), 3). In this case *τ*_*p*_ = 0 and Θ_*p*_ = 8 but the LOO and KOI TE-VIM modes are not equivalent (see Table 1). Under the KOI mode, some importance is assigned to *X*_4_ due to its correlation with *X*_3_, also greater importance is assigned to *X*_1_ versus *X*_3_ due to the correlation of *X*_1_ with *X*_2_. The LOO mode assigns little importance to *X*_1_, *X*_3_ since they are correlated with *X*_2_, *X*_4_ respectively. Shapley values represent a compromise between these modes, but require 2^*p*^ = 64 TE-VIMs to be evaluated. For this reason we compared only the LOO and KOI modes in our simulation.

### 3.1 Results

**Figure 1:**
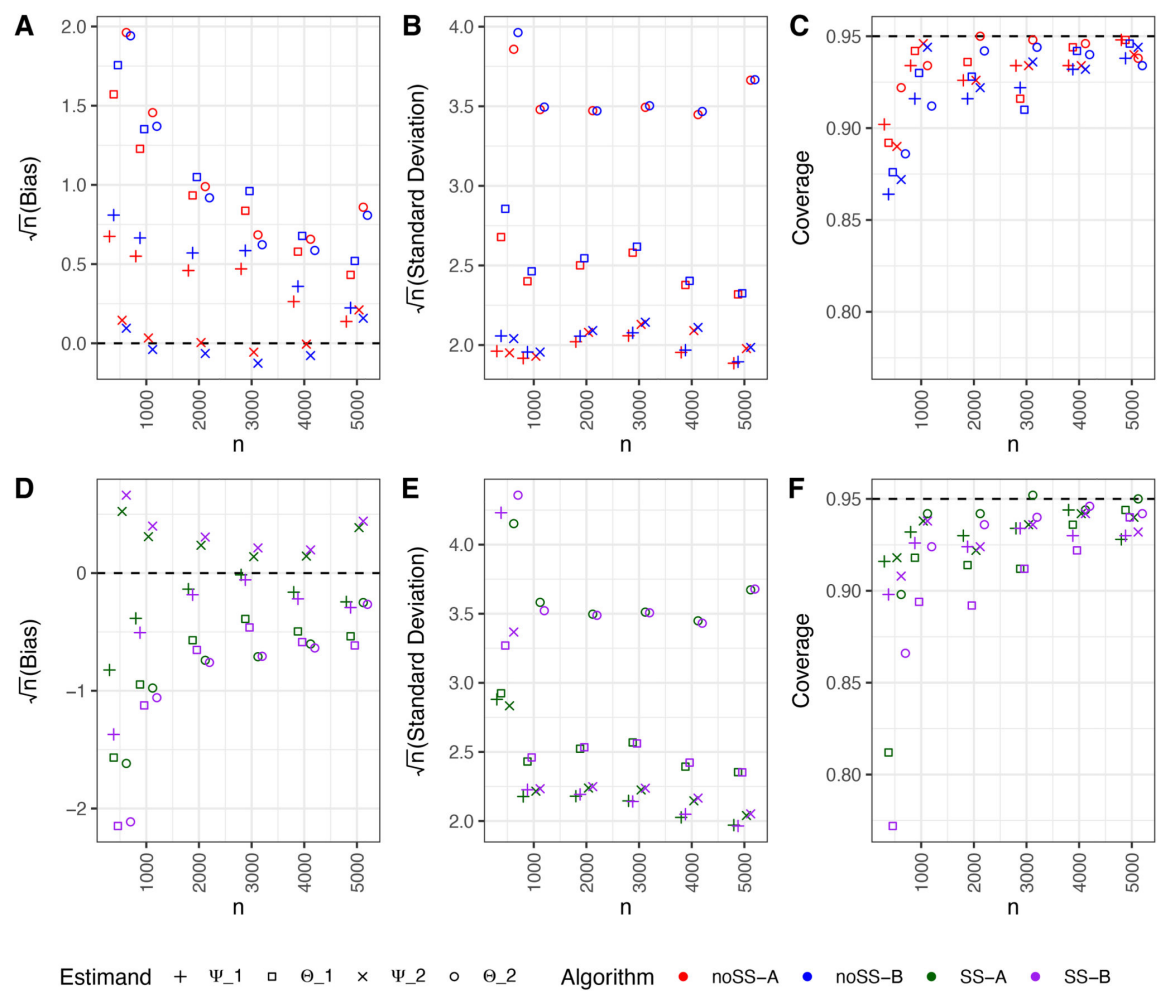
Bias (A, D), empirical standard deviation (B, E) and coverage (C, F) for estimators from DGP 1. Dashed lines indicate zero bias, and nominal 95% CI coverage. For readability, a small amount of ‘jitter’ has been added to the sample size, *n*. In order to present scaled and unscaled TE-VIMs together, the standard deviation and bias of scaled TE-VIMs has been multiplied by the true VTE. Note that the bias and variance scales differ between sub-plots A,B and D,E.

For each dataset, Θ_*s*_ and Ψ_*s*_ were estimated, along with their standard errors and Wald based (95%) confidence intervals (CIs). For DGPs 1 and 2, we also report the empirical probability that TE-VIMs correctly rank *X*_2_ as more important than *X*_1_. Figure 1 shows bias, variance, and coverage plots for DGP 1. Additional plots for DGP 2 and DGP 3 are in Supplement C.

For all DGPs, we see that TE-VIM estimators which do not use sample splitting tend to overestimate TE-VIMs (positive bias), whilst sample splitting estimators tend to underestimate TE-VIMs (negative bias). For DGP 1, we observe that, in small samples, DR-learner based algorithms (-B) produce larger bias, variance, and reduced CI coverage, than their T-learner counterparts (-A). Moreover, the scaled TE-VIM estimators tend to have smaller bias and variance than TE-VIM estimators. This trend appears reversed in the low-heterogeneity regime (DGP 2) when sample splitting is used. We believe this is due to extreme inverse weighting in the VTE estimate ${\hat{\Theta }}_{p}$, which appears in the denominator of ${\hat{\Psi }}_{1}$ and ${\hat{\Psi }}_{2}$.

For DGP 1, all algorithms recover the correct ranking with a high degree of accuracy. For DGP 2, this accuracy is reduced and conclusions based on scaled and unscaled TE-VIMs do not always agree, with the latter generally being more correct when cross-fitting is used (see Supplement C). For a given dataset, the ranking of scaled and unscaled TE-VIMs differs only when ${\hat{\Theta }}_{p}<0$. Therefore, we recommend that scaled TE-VIMs are only used when sensible VTE estimates are obtained, though TE-VIMs are also scientifically less relevant when there is little heterogeneity to account for.

In DGP 3 we observe that null importance does not seem to affect estimator bias, but does lead to reduced estimator standard deviations, as expected from theory, and decreased CI coverage. This phenomenon is especially clear when examining covariate *X*_4_, which has, in truth, null importance under the LOO mode, but not under the KOI mode. For the *X*_4_ LOO TE-VIM estimators we observe low variance and low CI coverage, whereas for the *X*_4_ KOI TE-VIM estimators we see higher variance and closer to nominal coverage.

## 4 Applied example: AIDS clinical trial

The AIDS Clinical Trials Group Protocol 175 (ACTG175) (Hammer et al., 1996) considers 2139 HIV patients with CD4 T-cell count between 200 and 500*mm*^−3^ randomized to 4 treatment groups: (i) zidovudine (ZDV); (ii) didanosine (ddI); (iii) ZDV+ddI; (iv) ZDV+zalcitabine. We compare groups (ii) and (iii) (*A* = 0, 1), with 561 and 522 patients respectively. We consider CD4 count at 20±5 weeks as a continuous outcome *Y* and 12 baseline covariates, 5 continuous: age, weight, Karnofsky score, CD4/CD8 count; and 7 binary: sex, homosexual activity (y/n), race (white/non-white), symptomatic (y/n), intravenous drug use history(y/n), hemophilia (y/n), and antiretroviral history (experienced/naive).

TE-VIMs for each covariates were estimated using all algorithms with *K* = 10 folds (around 10 folds is typical for cross-fitting procedures). A constant propensity score of 522/1083 ≈ 0.48 was used, since treatment is randomized. Fitted models for the outcome and CATEs were obtained using the ‘discrete’ Super Learner (van der Laan et al., 2007), an ensemble learning method, which selects the regression algorithm in a ‘learner library’ that minimizes some cross-validated risk. We used the SuperLearner R package implementation of this algorithm with 10 cross-validation folds, mean-squared-error loss, and a learner library containing various routines (glm, glmnet, gam, xgboost, ranger). Similar results are obtained when Super Learner regression is ablated and replaced with GAMs (see Supplement F).

**Figure 2:**
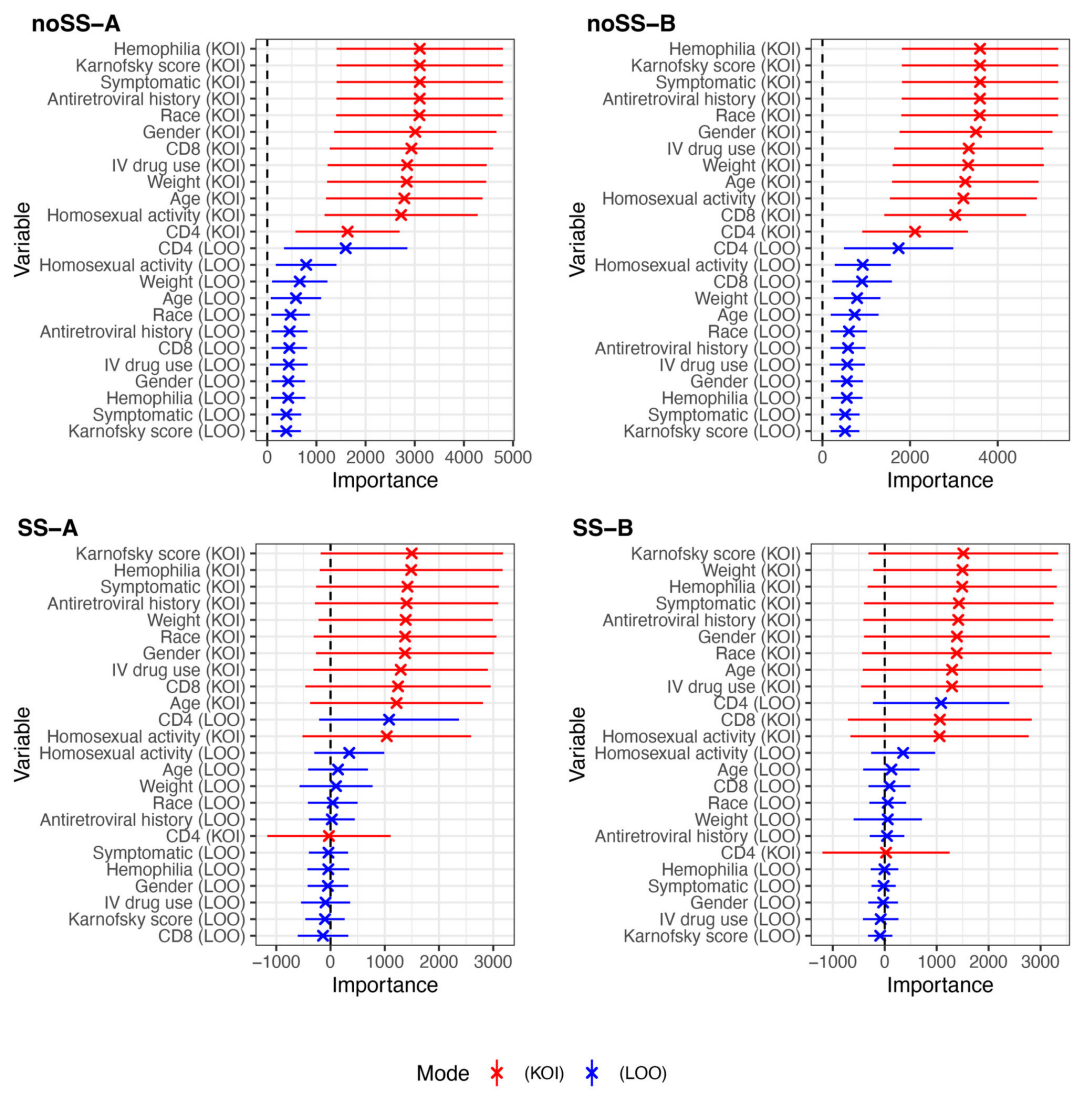
TE-VIM estimates ${\hat{\Theta }}_{s}$ from the ACTG175 study using each of the proposed Algorithms. Error bars indicate 95% CIs. In each plot, covariates are sorted according to their TE-VIM point estimate. Dashed lines indicate no importance. For the KOI mode, the TE-VIM represents the importance of the complement variable set, i.e. low-values denote high-importance of the KOI covariate.

**Figure 3:**
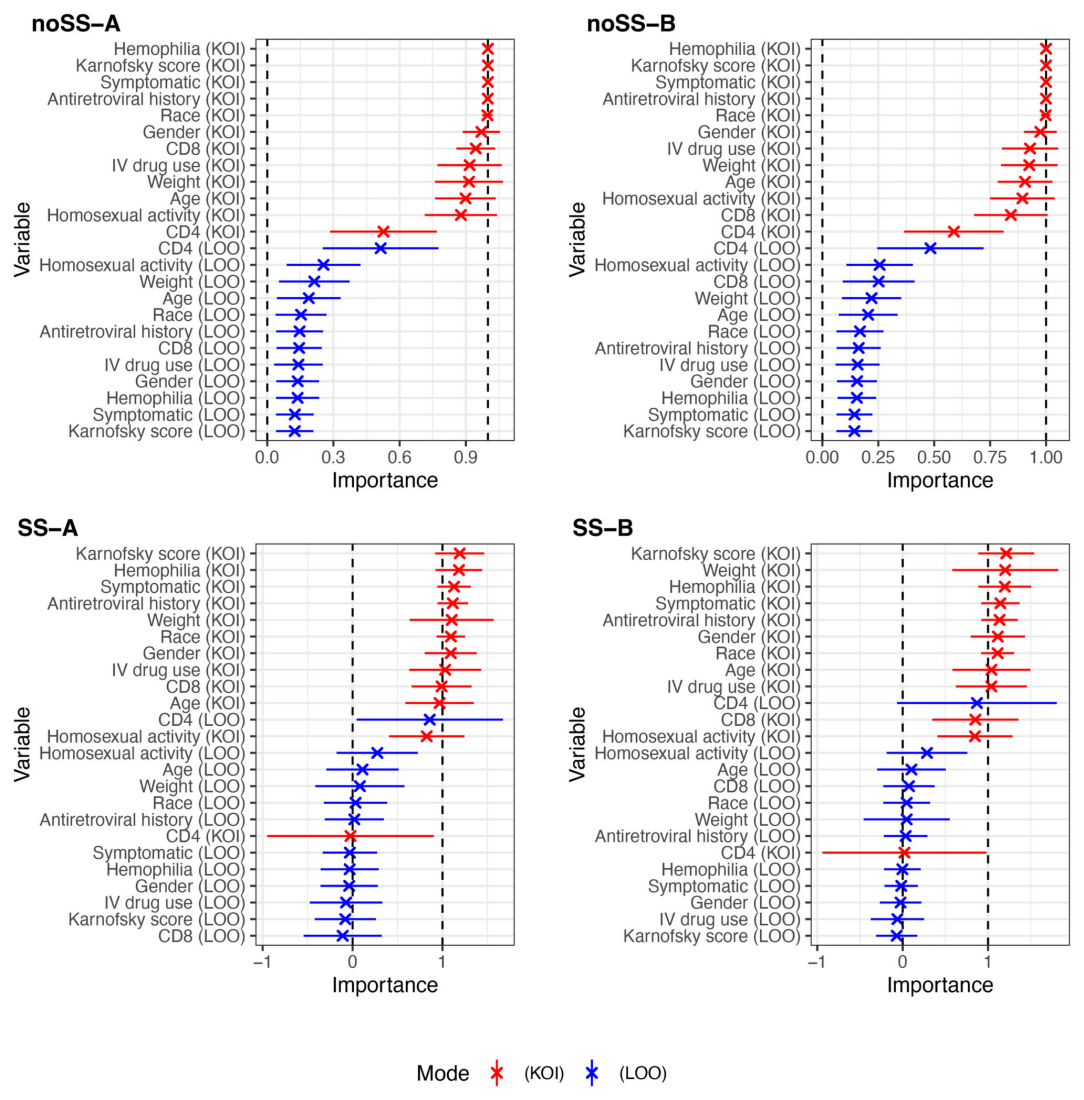
Scaled TE-VIM estimates ${\hat{\Psi }}_{s}$ from the ACTG175 study using each of the proposed Algorithms. Error bars indicate 95% CIs. In each plot, covariates are sorted according to their TE-VIM point estimate. Dashed lines indicate the [0, 1] support of the scaled TE-VIM. For the KOI mode, the TE-VIM represents the importance of the complement variable set, i.e. low-values denote high-importance of the KOI covariate.

AIPW estimates of the ATE using pseudo-outcomes from Algorithms noSS, SS-A, and SS-B were similar, respectively: 28.2*mm*^−3^ (CI: 14.0, 42.3; p<0.01); 28.4*mm*^−3^ (CI: 13.8, 42.9; p<0.01); 27.9*mm*^−3^ (CI: 13.3, 42.5; p<0.01), where all CIs are reported at 95% significance and p-values are of Wald type. VTE estimates differed substantially between algorithms with/without cross-fitting. With Algorithms noSS-A and -B returning estimates: 3100*mm*^−6^ (CI: 1410, 4790; p<0.01) and 3600*mm*^−6^ (CI: 1810, 5380; p<0.01), and for Algorithms SS-A and -B: 1260*mm*^−6^ (CI: -425, 2940; p=0.14) and 1250*mm*^−6^ (CI: -580, 3080; p=0.18). It is helpful to also consider the square root of the VTE estimates, which is on the same scale as the ATE. These are 55.7, 60.0, 35.5, and 35.3*mm*^−3^ for Algorithms noSS-A, -B, SS-A, and -B respectively. Based on the VTE CIs from Algorithms SS-A and -B, low treatment effect heterogeneity is a concern in this analysis. Figures 2 and 3 show unscaled and scaled TE-VIM estimates using LOO and KOI modes. All Algorithms rank CD4 count and homosexual activity as the most important covariates, with CD8 count also ranked highly by Algorithm SS-B under the KOI mode. We also observe (i) that standard errors are small for unimportant covariates, as expected due to the importance testing issues in Section 2.3.2; (ii) Algorithms SS-A and -B produce scaled TE-VIMs point estimates outside of (0, 1) more often than noSS-A and -B; and (iii) Algorithms noSS-A and -B do not produce scaled or unscaled TE-VIM point estimates with 95% CIs overlapping zero.

## 5 Related work and extensions

Here we discuss VIMs for the OTR and TE-VIMs for continuous treatments. Discussion on treatment effect scales, linear CATE projections (Boileau et al., 2023) and treatment effect cumulative distribution functions (Levy and van der Laan, 2018) are in Supplement D.

### 5.1 Optimal treatment rule - VIMs

Although TE-VIMs capture the importance of variables in explaining the CATE, this should not be confused with the importance of those variables in determining the OTR *d*(***x***) ≡ 𝕀{*τ* (***x***) > 0}, where 𝕀(.) is an indicator and, w.l.o.g., a greater outcome is preferred. For instance, treatment could be uniformly beneficial, in which case the OTR is to always treat, despite possible treatment effect heterogeneity. In such settings, analysis using TE-VIMs may provide useful insight to further improve therapies. Faced with OTR heterogeneity, one might consider nonparametric VIMs related to the OTR, e.g. by considering the risk *f* ↦ − *E*{*Y*^*f*(*X*)^}, where *f* : ℝ^*p*^ ↦ {0, 1} is a policy. This risk implies the OTR-VIM ${\text{Γ}}_{s}^{*}\equiv E\left\{Y^{d(X)}-Y^{d_{s}^{*}(X)}\right\}\geq 0\;$ where $d_{s}^{*}(x)\equiv I\left\{\tau_{s}(x)>0\right\}$ is the OTR given ***X***_−*s*_ (Zhang et al., 2015; Williamson et al., 2023). Thus, OTR-VIMs compare the OTR with suboptimal policies that are optimal within a restricted set of policies.

Under standard causal assumptions (consistency, positivity, exchangeability), the OTR-VIM is identified by ${\text{Γ}}_{s}^{*}=E\left[\tau (X)\left\{d(X)-d_{s}^{*}(X)\right\}\right]$. Unlike TE-VIMs, ${\text{Γ}}_{s}^{*}$ is not pathwise differentiable without additional assumptions e.g. that the OTR is insensitive to small changes in *P*_0_ (Luedtke and van der Laan, 2016), with the pathwise derivative usually used to construct efficient estimators. Analogous to Θ_*s*_ and Ω_*s*_, one could alternatively define OTR-VIMs using the risk *f* ↦ *E*[{*d*(***X***) − *f*(***X***)}^2^], which implies the VIM Γ_*s*_ ≡ *E*[*d*_*s*_(***X***){1 − *d*_*s*_(***X***)}] where *d*_*s*_(***x***) ≡ *E*{*d*(***X***)|***X***_−*s*_ = ***x***_−*s*_} = *Pr*{*τ* (***X***) > 0|***X***_−*s*_ = ***x***_−*s*_}. Note that *d*(***X***) ∈ {0, 1} and *d*_*s*_(***X***) ∈ [0, 1]. Like Θ_*s*_ and Ω_*s*_, Γ_*s*_ ∈ [0, 0.25] is invariant to linear transformations of the outcome, however, like ${\text{Γ}}_{s}^{*}$, additional assumptions on the OTR are required for pathwise differentiability of Γ_*s*_.

### 5.2 Continuous treatments

Continuous analogues of the CATE based on linear model projections are proposed by Hines et al. (2023). In particular, λ(***x***) ≡ cov(*A, Y* |***X*** = ***x***)/var(*A*|***X*** = ***x***) is well defined when *A* is continuous, and identifies the CATE under standard causal assumptions (consistency, positivity, exchangeability) when *A* is binary. Appealing to the risk *f* ↦ ‖λ(***X***) − *f*(***X***)‖^2^, one might extend the ATE, VTE, and TE-VIMs to continuous exposures using the estimands: E{λ(***X***)}, var{λ(***X***)}, and E[var{λ(***X***)|***X***_−*s*_}]/var{λ(***X***)}, which identify their CATE counterparts when *A* is binary. ICs for these estimands are obtained by replacing the pseudo-outcome *φ*(***z***) in the current work, with

$$\left[y-\mu (x)-\lambda (x)\{a-\pi (x)\}]\frac{a-\pi (x)}{\mathrm{var}(A|X=x)}+\lambda (x\right)$$

which reduces to *φ*(***z***) when *A* is binary. See Supplement D.9 for details.

## 6 Conclusion

We propose TE-VIMs, which capture the importance of variable subsets in explaining the CATE, and complement the VTE as a global heterogeneity measure (Levy et al., 2021). We derive efficient TE-VIM estimators that are amenable to machine learning of working models, thus, unlike existing proposals using causal forests, are not tied to specific algorithms (Athey et al., 2019). In studies where heterogeneous treatment effects are of primary interest, we recommend using TE-VIMs to select stratification variables for subgroup analyses, to support variable selection decisions for CATE/OTR models, or to rank effect modifiers. In studies where the main goal is to estimate the ATE, we recommend that VTE inference should also form part of the analysis, since the ATE and VTE can be used to inform on the marginal probability of adverse CATEs (see Supplement D.8). TE-VIMs may then form part of a secondary analysis when large treatment effect heterogeneity cannot be ruled out. We recommend that domain specific knowledge is used to select potential effect modifiers for VTE and TE-VIM analyses. We have outlined several frameworks for choosing the sets of variables to be included for the comparisons needed in the TE-VIM algorithm, such as leave-one-out or keep-one-in. Finally, we caution users against over-interpreting TE-VIM p-values due to the difficulty of testing the zero importance null and due to the multiplicity problem when the number of covariate subsets is large. The latter problem may be some-what attenuated using a Bonferroni approach. Any suggested covariate that may explain heterogeneity of treatment effects should be confirmed using independent data.

## Supplementary Material

Supplement

## Figures and Tables

**Table 1:** True TE-VIM values for DGP 3. For the KOI mode we report Θ_*p*_ − Θ_*s*_. Scaled TE-VIMs are obtained by dividing each value by Θ_*p*_ = 8. The Shapley values sum to Θ_*p*_ by design.

Target covariate	Leave-one-out	Keep-one-in	Shapley
*X*_1_	0.75	4	3.375
*X*_2_	3	6.25	4.625
*X*_3_	0.75	1	0.875
*X*_4_	0	0.25	0.125
*X*_5_	0	0	0
*X*_6_	0	0	0

## Data Availability

AIDS Clinical Trials Group Protocol 175 (ACTG175) data Hammer et al. (1996) is available on CRAN at https://CRAN.R-project.org/package=speff2trial.
