# Supplementary material for: Variable importance measures for heterogeneous causal effects
Source: Biometrics. Author manuscript; Available in PMC 2026 Mar 5. (PMC7618827; doi:10.1093/biomtc/ujaf140)
Supplement: Supplement [file EMS212112-supplement-Supplement.pdf]

Supplementary Materials

Web Appendices (referenced in Sections 1, 2 and 5) and Additional Figures (referenced in Sections 3 and 4) are available with this paper. Replication code is available at `https://github.com/ohines/tevims`.

Supplement

A Derivation of Efficient Influence Curve

We adopt the IC derivation formalism given in Hines et al. (2022). Specifically we let P_0 denote the true distribution of $(Y, A, \boldsymbol{X})$ and let $\tilde{P}$ denote a point mass at $(\tilde{y}, \tilde{a}, \tilde{\boldsymbol{x}})$. We further denote the parametric submodel $P_t = t\tilde{P} + (1-t)P_0$ where $t \in [0,1]$ is a scalar parameter, and we let ∂_t denote an operator such that for some function of $f(t)$, $\partial_t f(t) \equiv \frac{df(t)}{dt}|_{t=0}$.

Our goal is to derive an IC for $L^*_{P_0}\{h\} \equiv E_{P_0}[\{\tau(\boldsymbol{X}) - h(\boldsymbol{X})\}^2]$, for a known function $h : \mathbb{R}^p \mapsto \mathbb{R}$, which we will connect to ICs for Θ_s and Ψ_s.

We make use of the following lemma, which we demonstrate later in the proof. Letting $g_P(X)$ denote some functional of P, then

$$\begin{aligned}
&\partial_t E_{P_t}\{g_{P_t}(\boldsymbol{X})|\boldsymbol{X}_{-s} = x_{-s}\} \\
&= \frac{\tilde{f}(\boldsymbol{x}_{-s})}{f(\boldsymbol{x}_{-s})}\left[g_{P_0}(\tilde{\boldsymbol{x}}) - E_P\{g_{P_0}(\boldsymbol{X})|\boldsymbol{X}_{-s} = \boldsymbol{x}_{-s}\}\right] + E_{P_0}\{\partial_t g_{P_t}(\boldsymbol{X})|\boldsymbol{X}_{-s} = \boldsymbol{x}_{-s}\} \qquad (4)
\end{aligned}$$

where $\tilde{f}(.)$ and $f(.)$ denote the marginal 'densities' of $\boldsymbol{X}_{-s}$ under $\tilde{P}$ and P_0 respectively, which are w.l.o.g. absolutely continuous w.r.t. to a dominating measure. In practice this expression means that for discrete $\boldsymbol{X}_{-s}$ then $f(.)$ is a probability mass function and $\tilde{f}(.)$ is an indicator function. Similarly for continuous $\boldsymbol{X}_{-s}$ then $f(.)$ is a probability density function and $\tilde{f}(.)$ is a Dirac delta function. In both cases $\tilde{f}(\boldsymbol{x}_{-s})$ is a probability point mass, which is zero when $\tilde{\boldsymbol{x}}_{-s} \neq \boldsymbol{x}_{-s}$.

It follows immediately from (4) that,

$$\partial_t E_{P_t}\{g_{P_t}(\boldsymbol{X})\} = g_{P_0}(\tilde{\boldsymbol{x}}) - E_{P_0}\{g_{P_0}(\boldsymbol{X})\} + E_{P_0}\{\partial_t g_{P_t}(\boldsymbol{X})\} \qquad (5)$$

For the function $g_{P_t}(\boldsymbol{x}) = \{\tau_t(\boldsymbol{x}) - h(\boldsymbol{x})\}^2$, where $\tau_t(\boldsymbol{x})$ represents $\tau(\boldsymbol{x})$ under P_t, we obtain

$$\partial_t L^*_{P_t}\{h\} = \{\tau(\tilde{\boldsymbol{x}}) - h(\tilde{\boldsymbol{x}})\}^2 - L^*_{P_0}\{h\} + 2E_{P_0}[\{\tau(\boldsymbol{x}) - h(\boldsymbol{x})\}\partial_t \tau_t(\boldsymbol{x})] \qquad (6)$$

we use (4) and the fact that $\tau(\boldsymbol{x}) = \mu(1, \boldsymbol{x}) - \mu(0, \boldsymbol{x})$ to show that,

$$\partial_t \tau_t(\boldsymbol{x}) = \frac{\tilde{f}(\boldsymbol{x})}{f(\boldsymbol{x})}\{\tilde{y} - \mu(\tilde{a}, \boldsymbol{x})\}\frac{\tilde{a} - \pi(\boldsymbol{x})}{\pi(\boldsymbol{x})\{1 - \pi(\boldsymbol{x})\}}$$

hence we obtain the IC

$$\begin{aligned}
\partial_t L^*_{P_t}\{h\} &= \{\tau(\tilde{\boldsymbol{x}}) - h(\tilde{\boldsymbol{x}})\}^2 - L^*_{P_0}\{h\} + 2\{\tau(\tilde{\boldsymbol{x}}) - h(\tilde{\boldsymbol{x}})\}\{\tilde{y} - \mu(\tilde{a}, \tilde{\boldsymbol{x}})\}\frac{\tilde{a} - \pi(\tilde{\boldsymbol{x}})}{\pi(\tilde{\boldsymbol{x}})\{1 - \pi(\tilde{\boldsymbol{x}})\}} \qquad (7) \\
&= \{\tau(\tilde{\boldsymbol{x}}) - h(\tilde{\boldsymbol{x}})\}^2 - L^*_{P_0}\{h\} + 2\{\tau(\tilde{\boldsymbol{x}}) - h(\tilde{\boldsymbol{x}})\}\{\varphi(\tilde{\boldsymbol{z}}) - \tau(\tilde{\boldsymbol{x}})\} \qquad (8)
\end{aligned}$$

Completing the square of the expression above gives

$$\partial_t L^*_{P_t}\{h\} = \{\varphi(\tilde{\boldsymbol{z}}) - h(\tilde{\boldsymbol{x}})\}^2 - \{\varphi(\tilde{\boldsymbol{z}}) - \tau(\tilde{\boldsymbol{x}})\}^2 - L^*_{P_0}\{h\} \tag{9}$$

When replicating this proof, it is useful to note that for an arbitrary function $w(\boldsymbol{x})$

$$E_{P_0}\left\{\frac{\tilde{f}(\boldsymbol{X})}{f(\boldsymbol{X})} w(\boldsymbol{X})\right\} = w(\tilde{\boldsymbol{x}})$$

A.1 Proof of Lemma in (4)

To demonstrate (4) we write the lefthand side as

$$\partial_t \int g_{P_t}(\boldsymbol{x}^*) dP_{t,\boldsymbol{X}_s|\boldsymbol{x}_{-s}}(\boldsymbol{x}^*_s) = \int g_P(\boldsymbol{x}^*) \partial_t dP_{t,\boldsymbol{X}_s|\boldsymbol{x}_{-s}}(\boldsymbol{x}^*_s) + \int \{\partial_t g_{P_t}(\boldsymbol{x}^*)\} dP_{\boldsymbol{X}_s|\boldsymbol{x}_{-s}}(\boldsymbol{x}^*_s)$$

where $dP_{t,\boldsymbol{X}_s|\boldsymbol{x}_{-s}}(.)$ is the conditional distribution of $\boldsymbol{X}_s$ given $\boldsymbol{X}_{-s} = \boldsymbol{x}_{-s}$ under the parametric submodel and $\boldsymbol{x}^*_{-s} = \boldsymbol{x}_{-s}$. The second integral on the righthand side recovers the final term in (4). Hence the lemma follows once we show that

$$\partial_t dP_{t,\boldsymbol{X}_s|\boldsymbol{x}_{-s}}(\boldsymbol{x}^*_s) = \frac{\tilde{f}(\boldsymbol{x}_{-s})}{f(\boldsymbol{x}_{-s})}\{d\tilde{P}_{\boldsymbol{X}_s}(\boldsymbol{x}^*_s) - dP_{\boldsymbol{X}_s|\boldsymbol{x}_{-s}}(\boldsymbol{x}^*_s)\}$$

To do so, let μ denote a dominating measure and write

$$\begin{aligned} dP_{t,\boldsymbol{X}_s|\boldsymbol{x}_{-s}}(\boldsymbol{x}^*_s) &= f_{t,\boldsymbol{X}_s|\boldsymbol{x}_{-s}}(\boldsymbol{x}^*_s) d\mu(\boldsymbol{x}^*_s) \\ &= \frac{f_{t,\boldsymbol{X}}(\boldsymbol{x}^*)}{f_{t,\boldsymbol{X}_{-s}}(\boldsymbol{x}_{-s})} d\mu(\boldsymbol{x}^*_s) \end{aligned}$$

where $f_{t,\boldsymbol{X}}(.)$ and $f_{t,\boldsymbol{X}_{-s}}(.)$ denote the marginal densities of $\boldsymbol{X}$ and $\boldsymbol{X}_{-s}$ under the parametric submodel, P_t, i.e. they are the Radon-Nikodym derivatives w.r.t. μ. Applying the quotient rule, we obtain

$$\partial_t dP_{t,\boldsymbol{X}_s|\boldsymbol{x}_{-s}}(\boldsymbol{x}^*_s) = \frac{1}{f_{\boldsymbol{X}_{-s}}(\boldsymbol{x}_{-s})}\left[\partial_t f_{t,\boldsymbol{X}}(\boldsymbol{x}^*) - \frac{f_{\boldsymbol{X}}(\boldsymbol{x}^*)}{f_{\boldsymbol{X}_{-s}}(\boldsymbol{x}_{-s})}\partial_t f_{t,\boldsymbol{X}_{-s}}(\boldsymbol{x}_{-s})\right] d\mu(\boldsymbol{x}^*_s)$$

We now evaluate the derivative parts. Since $\partial_t P_t = \tilde{P} - P$, the marginal density derivatives will have a similar structure, as shown in the first expression below, where $f_{\boldsymbol{X}}(.)$ and $\tilde{f}_{\boldsymbol{X}}(.)$ denote marginal densities of $\boldsymbol{X}$ under $\tilde{P}$ and P, with likewise for $\boldsymbol{X}_{-s}$

$$\partial_t dP_{t,\boldsymbol{X}_s|\boldsymbol{x}_{-s}}(\boldsymbol{x}^*) = \frac{1}{f_{\boldsymbol{X}_{-s}}(\boldsymbol{x}_{-s})}\left[\{\tilde{f}_{\boldsymbol{X}}(\boldsymbol{x}^*) - f_{\boldsymbol{X}}(\boldsymbol{x}^*)\} - \frac{f_{\boldsymbol{X}}(\boldsymbol{x}^*)}{f_{\boldsymbol{X}_{-s}}(\boldsymbol{x}_{-s})}\{\tilde{f}_{\boldsymbol{X}_{-s}}(\boldsymbol{x}_{-s}) - f_{\boldsymbol{X}_{-s}}(\boldsymbol{x}_{-s})\}\right] d\mu(\boldsymbol{x}^*_s)$$

Since $\tilde{P}$ is a point mass, $\tilde{f}_{\boldsymbol{X}}(\boldsymbol{x}^*) = \tilde{f}_{\boldsymbol{X}_s}(\boldsymbol{x}_s^*)\tilde{f}_{\boldsymbol{X}_{-s}}(\boldsymbol{x}_{-s}^*)$. Also $\boldsymbol{x}_{-s}^* = \boldsymbol{x}_{-s}$ hence,

$$\begin{aligned}
\partial_t dP_{t,\boldsymbol{X}_s|\boldsymbol{x}_{-s}}(\boldsymbol{x}^*) &= \frac{\tilde{f}_{\boldsymbol{X}_{-s}}(\boldsymbol{x}_{-s})}{f_{\boldsymbol{X}_{-s}}(\boldsymbol{x}_{-s})}\left[\tilde{f}_{\boldsymbol{X}_s}(\boldsymbol{x}_s^*) - \frac{f_{\boldsymbol{X}}(\boldsymbol{x}^*)}{f_{\boldsymbol{X}_{-s}}(\boldsymbol{x}_{-s})}\right] d\mu(\boldsymbol{x}_s^*) \\
&= \frac{\tilde{f}_{\boldsymbol{X}_{-s}}(\boldsymbol{x}_{-s})}{f_{\boldsymbol{X}_{-s}}(\boldsymbol{x}_{-s})}\left[\tilde{f}_{\boldsymbol{X}_s}(\boldsymbol{x}_s^*) - f_{\boldsymbol{X}_s|\boldsymbol{x}_{-s}}(\boldsymbol{x}_s^*)\right] d\mu(\boldsymbol{x}_s^*)
\end{aligned}$$

Thus, the result follows.

B Estimator Asymptotic Distributions

In this Appendix we use a common empirical processes notation, where we define linear operators P_0 and $\mathbb{P}_n$ such that for some function $h(\boldsymbol{Z})$, $P_0\{h(\boldsymbol{Z})\} \equiv E\{h(\boldsymbol{Z})\}$ and $\mathbb{P}_n\{h(\boldsymbol{Z})\} \equiv n^{-1}\sum_{i=1}^n h(\boldsymbol{z}_i)$. To simplify notation we also largely omit function arguments, for example $\tau = \tau(\boldsymbol{X})$ with similar for $\hat{\tau}, \tau_s, \hat{\tau}_s, \pi, \hat{\pi}, \hat{\varphi}$.

B.1 Proof of Theorem 1

Define

$$\begin{aligned}
\hat{\varphi}(\boldsymbol{z}) &\equiv \{y - \hat{\mu}(a,\boldsymbol{x})\}\frac{a - \hat{\pi}(\boldsymbol{x})}{\hat{\pi}(\boldsymbol{x})\{1-\hat{\pi}(\boldsymbol{x})\}} + \hat{\mu}(1,\boldsymbol{x}) - \hat{\mu}(0,\boldsymbol{x}) \\
\hat{\phi}_s(\boldsymbol{z}) &\equiv \{\hat{\varphi}(\boldsymbol{z}) - \hat{\tau}_s(\boldsymbol{x})\}^2 - \{\hat{\varphi}(\boldsymbol{z}) - \hat{\tau}(\boldsymbol{x})\}^2 - \hat{\Theta}_s^0
\end{aligned}$$

where $\hat{\Theta}_s^0$ is an initial estimate of Θ_s. Without making any restrictions we write

$$\hat{\Theta}_s - \Theta_s = (\mathbb{P}_n - P_0)\{\phi_s(\boldsymbol{Z})\} + R_n + H_n \tag{10}$$

$$\hat{\Theta}_s \equiv \hat{\Theta}_s^0 + \mathbb{P}_n\{\hat{\phi}_s(\boldsymbol{Z})\} \tag{11}$$

$$R_n \equiv \hat{\Theta}_s^0 - \Theta_s + P_0\{\hat{\phi}_s(\boldsymbol{Z})\} \tag{12}$$

$$H_n \equiv (\mathbb{P}_n - P_0)\{\hat{\phi}_s(\boldsymbol{Z}) - \phi_s(\boldsymbol{Z})\}. \tag{13}$$

We will show that the remainder term $R_n = o_P(n^{-1/2})$ and the empirical process term $H_n = o_P(n^{-1/2})$, and hence the result follows since $P_0\{\phi_\psi(\boldsymbol{Z})\} = 0$.

B.2 The remainder term

Evaluating the remainder $R_n = E\{\hat{\phi}_s(\boldsymbol{Z}) + \hat{\Theta}_s^0 - \Theta_s\}$ gives

$$R_n = E\left[\{\hat{\varphi} - \hat{\tau}_s\}^2 - \{\hat{\varphi} - \hat{\tau}\}^2 - \{\tau - \tau_s\}^2\right]$$

where we have used the fact that $\Theta_s = E[\{\tau - \tau_s\}^2]$. By algebraic manipulation, we write

$$R_n = E\left[\{\hat{\tau} - \hat{\tau}_s\}^2 - \{\tau - \tau_s\}^2 + 2\{\hat{\tau} - \hat{\tau}_s\}\{\hat{\varphi} - \hat{\tau}\}\right]$$

We then use the identity,

$$E\left[\{\hat{\tau} - \hat{\tau}_s\}^2 - \{\tau - \tau_s\}^2\right] = E\left[\{\tau_s - \hat{\tau}_s\}^2 - \{\tau - \hat{\tau}\}^2 + 2\{\hat{\tau} - \hat{\tau}_s\}\{\hat{\tau} - \tau\}\right]$$

to rewrite the remainder term as the sum of two error terms,

$$\begin{aligned} R_n &= E\left[\{\hat{\tau} - \hat{\tau}_s\}^2 - \{\tau - \tau_s\}^2 + 2\{\hat{\tau} - \hat{\tau}_s\}\{\hat{\varphi} - \hat{\tau}\}\right] \\ &= E\left[\{\tau_s - \hat{\tau}_s\}^2 - \{\tau - \hat{\tau}\}^2 + 2\{\hat{\tau} - \hat{\tau}_s\}\{\hat{\tau} - \tau\} + 2\{\hat{\tau} - \hat{\tau}_s\}\{\hat{\varphi} - \hat{\tau}\}\right] \\ &= E\left[\{\tau_s - \hat{\tau}_s\}^2 - \{\tau - \hat{\tau}\}^2 + 2\{\hat{\tau} - \hat{\tau}_s\}\{\hat{\varphi} - \tau\}\right] \\ &= \underbrace{E\left[\{\tau_s - \hat{\tau}_s\}^2 - \{\tau - \hat{\tau}\}^2\right]}_{\text{CATE error}} + \underbrace{2E\left[\{\hat{\tau} - \hat{\tau}_s\}r\right]}_{\text{Pseudo-outcome error}} \end{aligned}$$

where $r = r(\boldsymbol{X})$ is defined by $r(\boldsymbol{x}) \equiv E\left[\hat{\varphi}|\boldsymbol{X} = \boldsymbol{x}\right] - \tau(\boldsymbol{x})$, which represents a pseudo-outcome error in the sense that $r(\boldsymbol{x}) = E\left[\hat{\varphi} - \varphi|\boldsymbol{X} = \boldsymbol{x}\right]$. Splitting the remainder in to two error terms allows us to consider that the CATE error is $o_P(n^{-1/2})$ when (A2) holds. For the pseudo-outcome error we use the Cauchy-Schwarz inequality to show that

$$E\left[\{\hat{\tau} - \hat{\tau}_s\}r\right]^2 \le E\left[\{\hat{\tau} - \hat{\tau}_s\}^2\right] E\left[r^2\right] \le \delta^2 E\left[r^2\right]$$

Hence the pseudo-outcome error term is $o_P(n^{-1/2})$ if r is $o_P(n^{-1/2})$. By iterated expectation

$$r(\boldsymbol{x}) = \left\{\frac{\pi(\boldsymbol{x})}{\hat{\pi}(\boldsymbol{x})} - 1\right\}\{\mu(1,\boldsymbol{x}) - \hat{\mu}(1,\boldsymbol{x})\} - \left\{\frac{1 - \pi(\boldsymbol{x})}{1 - \hat{\pi}(\boldsymbol{x})} - 1\right\}\{\mu(0,\boldsymbol{x}) - \hat{\mu}(0,\boldsymbol{x})\}$$

Using the inequality $(a+b)^2 \le 2(a^2 + b^2)$ then

$$\begin{aligned} r^2(\boldsymbol{x}) &\le 2\left\{\frac{\pi(\boldsymbol{x})}{\hat{\pi}(\boldsymbol{x})} - 1\right\}^2 \{\mu(1,\boldsymbol{x}) - \hat{\mu}(1,\boldsymbol{x})\}^2 + 2\left\{\frac{1 - \pi(\boldsymbol{x})}{1 - \hat{\pi}(\boldsymbol{x})} - 1\right\}^2 \{\mu(0,\boldsymbol{x}) - \hat{\mu}(0,\boldsymbol{x})\}^2 \\ &\le \left(\frac{2}{\epsilon^2}\right)\{\pi(\boldsymbol{x}) - \hat{\pi}(\boldsymbol{x})\}^2 \left[\{\mu(1,\boldsymbol{x}) - \hat{\mu}(1,\boldsymbol{x})\}^2 + \{\mu(0,\boldsymbol{x}) - \hat{\mu}(0,\boldsymbol{x})\}^2\right] \end{aligned}$$

with the second inequality follows since $\hat{\pi} \in (\epsilon, 1-\epsilon)$. The final expression above is $o_P(n^{-1})$ under (A1), which completes the proof that R_n itself is $o_P(n^{-1/2})$.

B.3 The empirical process term

First write the empirical process term as the sum

$$\begin{aligned} H_n &= (\mathbb{P}_n - P_0)\{\Theta_s - \hat{\Theta}_s^0\} \\ &+ 2(\mathbb{P}_n - P_0)\{(\hat{\varphi} - \varphi)(\hat{\tau} - \hat{\tau}_s)\} \\ &+ (\mathbb{P}_n - P_0)\{(\varphi - \hat{\tau}_s)^2 - (\varphi - \tau_s)^2\} \\ &- (\mathbb{P}_n - P_0)\{(\varphi - \hat{\tau})^2 - (\varphi - \tau)^2\} \end{aligned}$$

Note that the first term is zero since $(\mathbb{P}_n - P_0)\{\Theta_s - \hat{\Theta}_s^0\} = (\Theta_s - \hat{\Theta}_s^0)(\mathbb{P}_n - P_0)\{1\} = 0$. When the Donsker condition holds, then, by Lemma 19.24 of , the second term is $o_P(n^{-1/2})$ provided (i) that $E\{(\hat{\varphi}-\varphi)^2(\hat{\tau}-\hat{\tau}_s)^2\} = o_p(1)$, the third term is $o_P(n^{-1/2})$ provided (ii) that $E\left[\left\{(\varphi - \hat{\tau}_s)^2 - (\varphi - \tau_s)^2\right\}^2\right] = o_p(1)$, and the fourth term is $o_P(n^{-1/2})$ provided (iii) that $E\left[\left\{(\varphi - \hat{\tau})^2 - (\varphi - \tau)^2\right\}^2\right] = o_p(1)$. Similarly, under sample splitting then by Chebyshev's inequality, (i), (ii), and (iii) are also sufficient conditions for H_n to be $o_P(n^{-1/2})$. We will examine these conditions in reverse order.

For (iii) we write

$$\begin{aligned} (\varphi - \hat{\tau})^2 - (\varphi - \tau)^2 &= 2(\varphi - \tau)(\tau - \hat{\tau}) + (\tau - \hat{\tau})^2 \\ E\left[\left\{(\varphi - \hat{\tau})^2 - (\varphi - \tau)^2\right\}^2 |\boldsymbol{X}\right] &= 4\text{var}(\varphi|\boldsymbol{X})(\tau - \hat{\tau})^2 + (\tau - \hat{\tau})^4 \end{aligned}$$

Since $\hat{\tau}$ is consistent, and $\text{var}(\varphi|\boldsymbol{X}) < K$ then (iii) holds. Also since $\hat{\tau}_s$ is consistent then (ii) holds in the same way. For (i) we apply Hölder's inequality to obtain

$$\begin{aligned} E\{(\hat{\varphi} - \varphi)^2(\hat{\tau} - \hat{\tau}_s)^2\} &\leq E\{(\hat{\tau} - \hat{\tau}_s)^2\}||(\hat{\varphi} - \varphi)^2||_\infty \\ &\leq \delta^2||(\hat{\varphi} - \varphi)^2||_\infty \end{aligned}$$

where $||.||_\infty$ denotes the supremum norm. Since $\hat{\varphi}$ is a uniformly consistent estimator of φ, $||(\hat{\varphi} - \varphi)^2||_\infty = o_p(1)$, thus $H_n = o_P(n^{-1/2})$ which completes the proof.

B.4 Proof of Theorem 2

Suppose we have two regular asymptotically linear estimators

$$\hat{\Theta}_s - \Theta_s = \mathbb{P}_n\{\phi_s(\boldsymbol{Z})\} + o_p(n^{-1/2}) \tag{14}$$

$$\hat{\Theta}_p - \Theta_p = \mathbb{P}_n\{\phi_p(\boldsymbol{Z})\} + o_p(n^{-1/2}) \tag{15}$$

It follows by algebraic manipulations that

$$n^{1/2}(\hat{\Psi}_s - \Psi_s) = \frac{\Theta_p}{\hat{\Theta}_p}\left[n^{1/2}\mathbb{P}_n\{\Phi_s(\boldsymbol{Z})\} + o_p(1)\right]$$

where $\Phi_s(z) = \{\phi_s(z) - \Psi_s\phi_p(z)\}/\Theta_p$ is the IC of Ψ_s. By Slutsky's Theorem and the fact that $\hat{\Theta}_p/\Theta_p$ converges to 1 in probability

$$\lim_{n\to\infty} n^{1/2}(\hat{\Psi}_s - \Psi_s) = \lim_{n\to\infty} n^{1/2}\mathbb{P}_n\{\Phi_s(\boldsymbol{Z})\}$$

which gives the desired result due to the central limit theorem. We note that this set up is quite general when one considers estimands which are written as the ratio of two other estimands, such as Ψ_s in the present context.

Clearly (14) follows by Theorem 1. We must therefore check that (15) also holds.

Most of the steps in the Proof of Theorem 1 can be applied directly to Θ_p. When decomposing the empirical process term, however, we are left with the term

$$(\mathbb{P}_n - P_0)\{(\varphi - \hat{\tau}_p^*)^2 - (\varphi - \tau_p)^2\}$$

in place of the corresponding term involving τ_s. Since τ_p and $\hat{\tau}_p^*$ are constant this term reduces to

$$-2(\hat{\tau}_p^* - \tau_p)(\mathbb{P}_n - P_0)\{\varphi\}$$

Next we note that the conditions of Theorem 1 imply that the AIPW is a RAL estimator of the ATE

$$\hat{\tau}_p^* - \tau_p = \mathbb{P}_n\{\varphi - \tau_p\} + o_P(n^{-1/2})$$

See e.g. Theorem 5.1 of Chernozhukov et al. (2018). Also, $(\mathbb{P}_n - P_0)\{\varphi\} \xrightarrow{p} 0$ by the weak law of large numbers. Therefore this term in the empirical process term decomposition will be $o_P(n^{-1/2})$, which completes the proof.

C Additional plots for simulation results

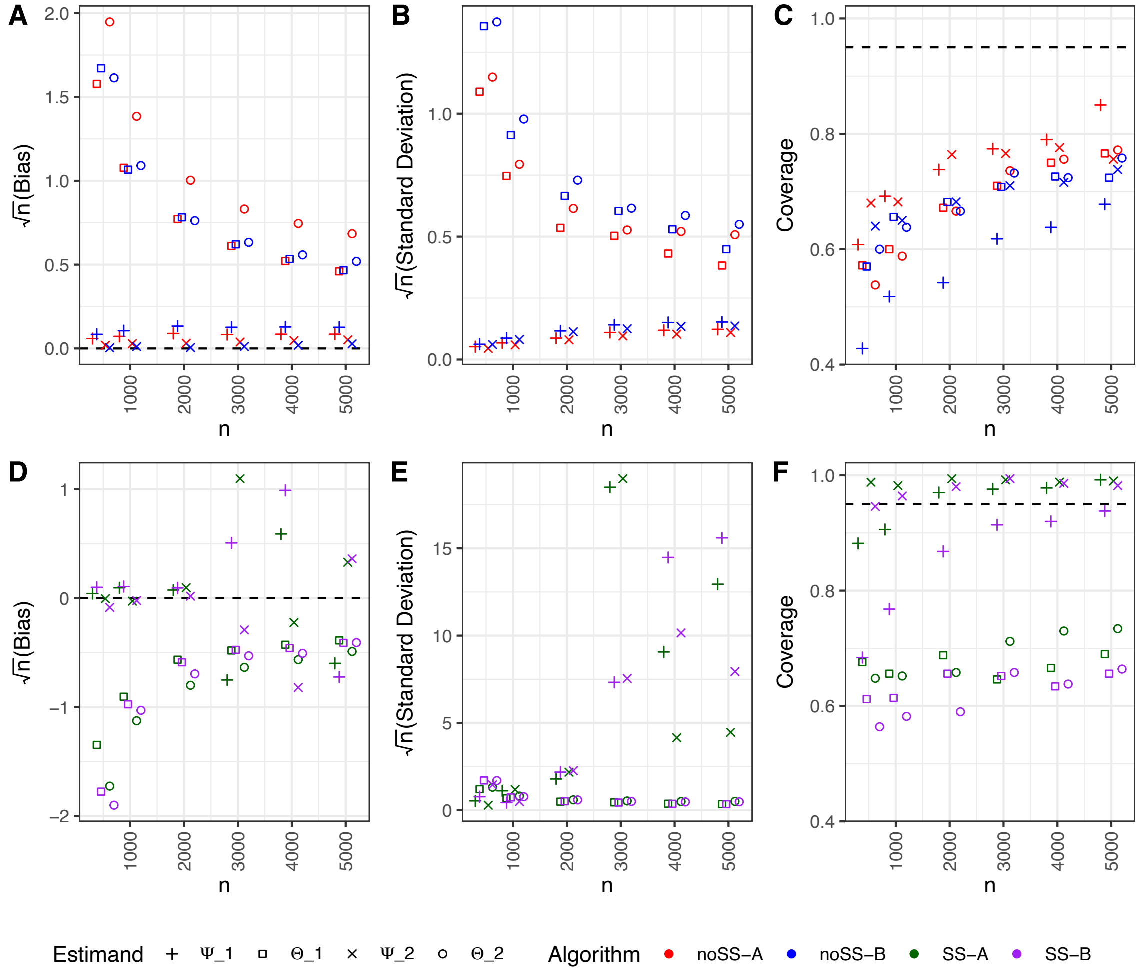

Figure 4: Bias (A, D), empirical standard deviation (B, E) and coverage (C, F) for estimators from DGP 2. Dashed lines indicate zero bias, and nominal 95% CI coverage. For readability, a small amount of 'jitter' has been added to the sample size, n. In order to present scaled and unscaled TE-VIMs together, the standard deviation and bias of scaled TE-VIMs has been multiplied by the true VTE. Note that the bias and variance scales differ between sub-plots A,B and D,E.

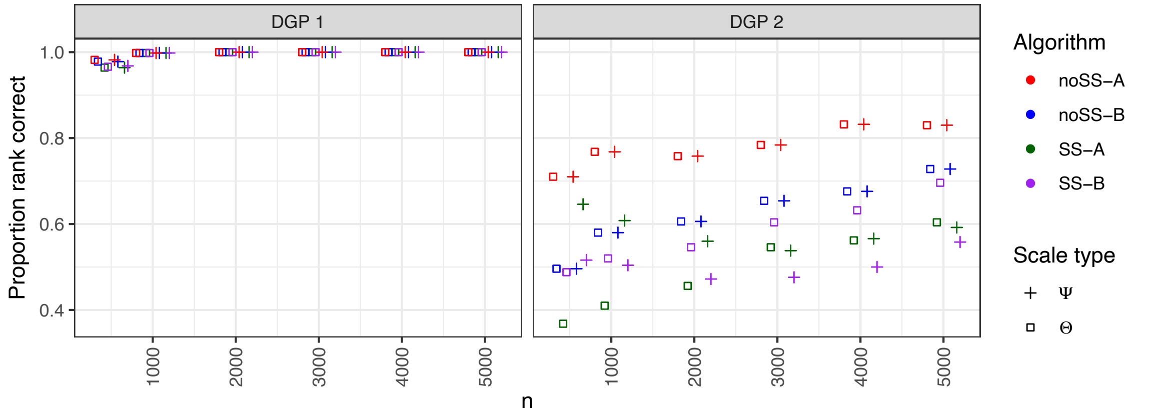

Figure 5: Empirical probability that (scaled) TE-VIMs recover the correct importance ranking for DGPs 1 and 2. For readability, a small amount of 'jitter' has been added to n.

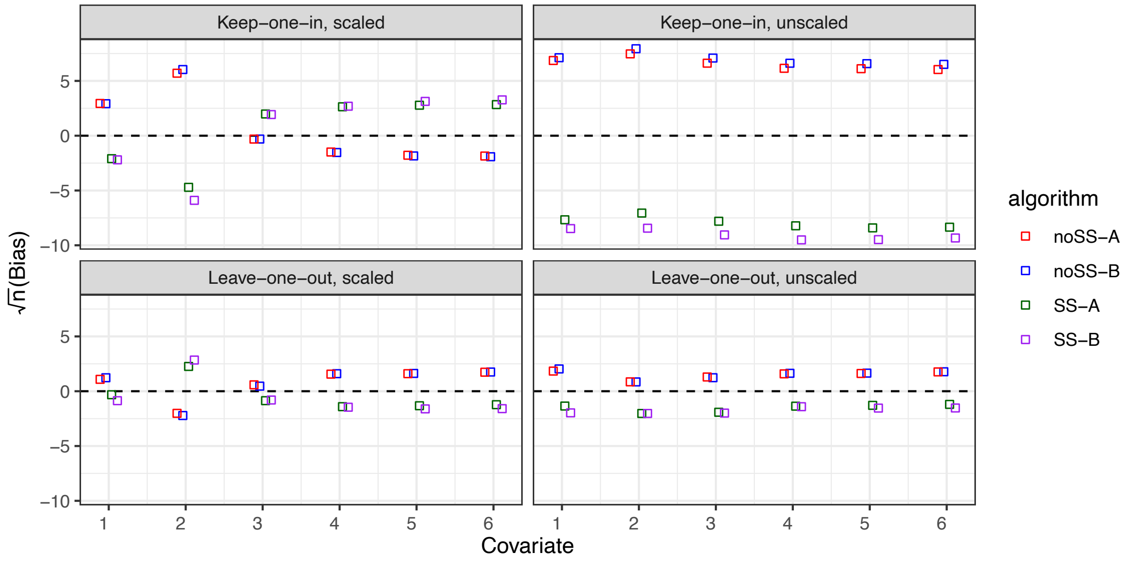

Figure 6: Empirical bias for estimators from DGP 3. Dashed lines indicate zero bias. In order to present scaled and unscaled TE-VIMs together, the bias of scaled TE-VIMs has been multiplied by the true VTE $\Theta_p = 8$. Here scaled and unscaled refers to estimators of the type Ψ and Θ respectively.

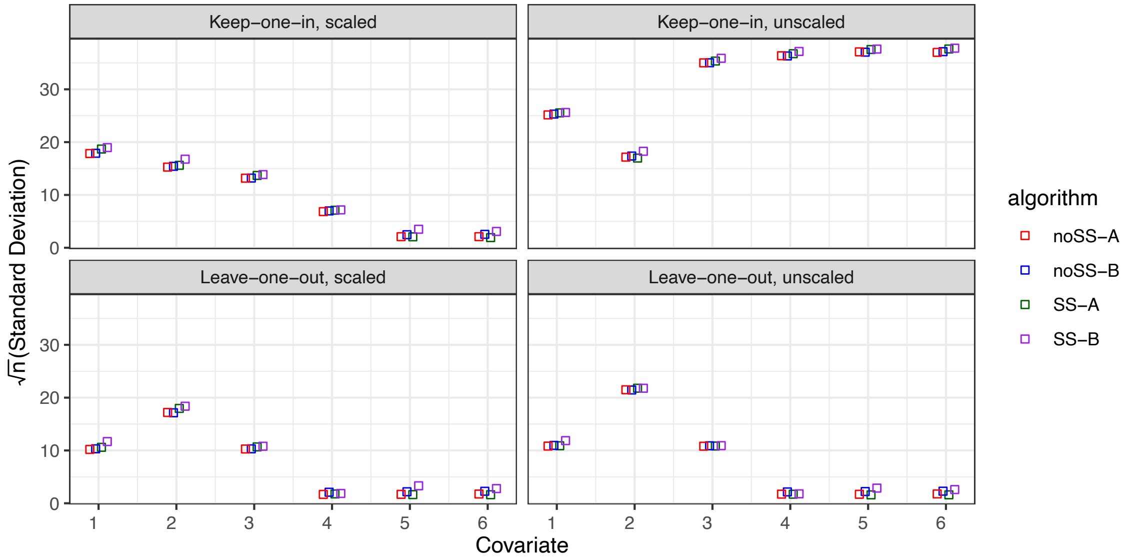

Figure 7: Empirical standard deviation of estimators from DGP 3. In order to present scaled and unscaled TE-VIMs together, the standard deviation of scaled TE-VIMs has been multiplied by the true VTE $\Theta_p = 8$. Here scaled and unscaled refers to estimators of the type Ψ and Θ respectively.

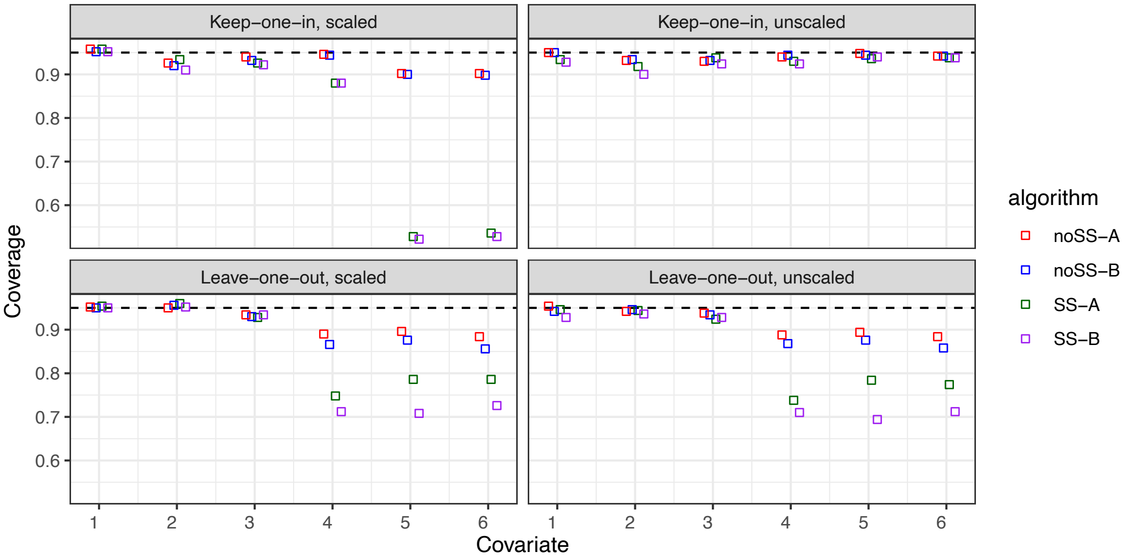

Figure 8: Empirical CI coverage for estimators from DGP 3. Dashed lines indicate nominal 95% CI coverage. Here scaled and unscaled refers to estimators of the type Ψ and Θ respectively.

D Additional discussion

D.1 Representations of TE-VIMs

We claim that the following representations of Θ_s are equivalent

$$\begin{aligned}\Theta_s &= E[\{\tau(\boldsymbol{X}) - \tau_s(\boldsymbol{X})\}^2] \\ &= E[\text{var}\{\tau(\boldsymbol{X})|\boldsymbol{X}_{-s}\}] \\ &= \text{var}\{\tau(\boldsymbol{X})\} - \text{var}\{\tau_s(\boldsymbol{X})\}\end{aligned}$$

Proof: The second line follows from the first by noting that $\tau_s(\boldsymbol{x}) = E\{\tau(\boldsymbol{X})|\boldsymbol{X}_{-s} = \boldsymbol{x}_{-s}\}$. The third line follows from the first since

$$\begin{aligned}\Theta_s &= E[\{\tau(\boldsymbol{X}) - \tau_s(\boldsymbol{X})\}^2] \\ &= E\{\tau^2(\boldsymbol{X})\} + E\{\tau_s^2(\boldsymbol{X})\} - 2E\{\tau(\boldsymbol{X})\tau_s(\boldsymbol{X})\} \\ &= E\{\tau^2(\boldsymbol{X})\} - E\{\tau_s^2(\boldsymbol{X})\} \\ &= \left[E\{\tau^2(\boldsymbol{X})\} - \tau_p^2\right] - \left[E\{\tau_s^2(\boldsymbol{X})\} - \tau_p^2\right] \\ &= \text{var}\{\tau(\boldsymbol{X})\} - \text{var}\{\tau_s(\boldsymbol{X})\}\end{aligned}$$

where we the law of iterated expectations to obtain $E\{\tau(\boldsymbol{X})\tau_s(\boldsymbol{X})\} = E\{E\{\tau(\boldsymbol{X})\tau_s(\boldsymbol{X})|\boldsymbol{X}_{-s}\}\} = E\{\tau_s^2(\boldsymbol{X})\}$.

D.2 Invariance to transformations

The scaled TE-VIM Ψ_s is invariant to linear outcome transformations. To see why, let $\tilde{Y} = bY + k$ and $\tilde{Y}^a = bY^a + k$ for constants $b \neq 0$ and k. Letting superscript tilde to denote the modified values,

$$\begin{aligned}&\tilde{\tau}(x) \equiv E(\tilde{Y}^1 - \tilde{Y}^0|\boldsymbol{X} = \boldsymbol{x}) = b\tau(\boldsymbol{x}) \\ \implies\ &\tilde{\Theta}_s \equiv E[\text{var}\{\tilde{\tau}(\boldsymbol{X})|\boldsymbol{X}_{-s}\}] = b^2\Theta_s \\ \implies\ &\tilde{\Psi}_s \equiv \frac{\tilde{\Theta}_s}{\tilde{\Theta}_p} = \frac{\Theta_s}{\Theta_p} = \Psi_s.\end{aligned}$$

Moreover, Ψ_s is invariant to invertible component wise mappings of $\boldsymbol{X}$. To see why, consider a mapping $g : \mathbb{R}^p \mapsto \mathbb{R}^p$ such that

$$g(\boldsymbol{x}) = (g_1(x_1), ..., g_p(x_p))$$

where $g_j : \mathbb{R} \mapsto \mathbb{R}$ is an invertible function for $j \in \{1, ..., p\}$. The claim follows since the conditional distribution of Y induced by $\boldsymbol{X}_{-s}$ is the same as that induced by $g(\boldsymbol{X})_{-s}$. In particular, using superscript tilde to denote a different set of modified values,

$$\begin{aligned}
&\tilde{\tau}(g(\boldsymbol{x})) \equiv E(Y^1 - Y^0 | g(\boldsymbol{X}) = g(\boldsymbol{x})) = \tau(\boldsymbol{x}) \\
\implies &\tilde{\Theta}_s \equiv E[\text{var}\{\tilde{\tau}(g(\boldsymbol{X}))|g(\boldsymbol{X})_{-s}\}] = E[\text{var}\{\tau(\boldsymbol{X})|g(\boldsymbol{X})_{-s}\}] = \Theta_s \\
\implies &\tilde{\Psi}_s \equiv \frac{\tilde{\Theta}_s}{\tilde{\Theta}_p} = \frac{\Theta_s}{\Theta_p} = \Psi_s.
\end{aligned}$$

D.3 Shapley values

We use the Shapley value definition given by Williamson and Feng (2020) for an arbitrary value/ loss function. For covariate $j \in \{1, ..., p\}$, and letting $\mathcal{P}_j$ denote the power set (set of all possible subsets) of $\{1, ..., p\} \setminus \{j\}$, we define the TE-VIM Shapley value

$$S_j \equiv \sum_{s \in \mathcal{P}_j} \frac{1}{p} \binom{p-1}{|s|}^{-1} \{\Theta_{s \cup \{j\}} - \Theta_s\}$$

Since $\Theta_{s \cup \{j\}} \geq \Theta_s$ for all s, then $S_j \geq 0$. Also, by construction

$$\Theta_p = \sum_{j=1}^{p} S_j.$$

D.4 Plug-in estimators

Plug-in estimators for the TE-VIM Θ_s and the scaled TE-VIM Ψ_s are non-trivial to construct using estimators for $\tau(\boldsymbol{x})$ and $\tau_s(\boldsymbol{x})$. We illustrate this by considering plug-in estimation of the regression-VIM Ω_s which suffers similar difficulties. Writing the regression-VIM as

$$\Omega_s = E\left[\{\mu(\boldsymbol{X}) - \mu({}_s\boldsymbol{X})\}^2\right]$$

Williamson et al. (2021) construct a 'naive plug-in' estimator

$$\hat{\Omega}_s^0 = n^{-1} \sum_{i=1}^{n} \{\hat{\mu}(\boldsymbol{x}_i) - \hat{\mu}({}_s\boldsymbol{x}_i)\}^2.$$

However, this estimator is not strictly plug-in in the estimand mapping sense discussed in the main paper. To see why, we write Ω_s in terms of $\mu(\boldsymbol{x})$, the distribution of $\boldsymbol{X}_s$ given

$\boldsymbol{X}_{-s}$, which we denote by the measure $dP_0(\boldsymbol{x}_s|\boldsymbol{x}_{-s})$, and the distribution of $\boldsymbol{X}_{-s}$, denoted $dP_0(\boldsymbol{x}_s)$,

$$\Omega_s = \int \left[\mu(\boldsymbol{x}) - \left\{ \int \mu(\boldsymbol{x}) \underbrace{dP_0(\boldsymbol{x}_s|\boldsymbol{x}_{-s})}_{\text{regression}} \right\} \right]^2 \underbrace{dP_0(\boldsymbol{x}_s|\boldsymbol{x}_{-s})}_{\text{empirical}} dP_0(\boldsymbol{x}_s).$$

Hence, in the estimand definition, the distribution of $\boldsymbol{X}_s$ given $\boldsymbol{X}_{-s}$ appears twice. However, the $\hat{\Omega}_s^0$ estimator effectively uses, two different (and possibly inconsistent) implicit distributions to approximate $dP_0(\boldsymbol{x}_s|\boldsymbol{x}_{-s})$. The first is implied by the regression estimate $\hat{\mu}_s(\boldsymbol{x})$, whilst the second is implied by the empirical distribution of covariates. In particular, this inconsistency means that there is no guarantee that

$$n^{-1} \sum_{i=1}^{n} \hat{\mu}(\boldsymbol{x}_i) = n^{-1} \sum_{i=1}^{n} \hat{\mu}_s(\boldsymbol{x}_i)$$

without additional steps taken to ensure that this identity holds e.g. through targeting (in a TMLE sense) an initial regression estimator of $\hat{\mu}_s(\boldsymbol{x})$.

We remark that for the Proofs in Appendix B, the exact form of the initial TE-VIM estimator $\hat{\Theta}_s^0$ does not affect the form of the final estimator $\hat{\Theta}_s$, which, can be thought of as a bias-corrected version of $\hat{\Theta}_s^0$. As such, one could let $\hat{\Theta}_s^0 = n^{-1} \sum_{i=1}^{n} \{\hat{\tau}(\boldsymbol{x}_i) - \hat{\tau}({}_s\boldsymbol{x}_i)\}^2$, though this estimand is also not plug-in for the reasons above.

D.5 TE-VIM estimation on different scales

The TE-VIM $\Theta_s \geq 0$ and the scaled TE-VIM $\Psi_s \in [0, 1]$ are both bounded, therefore one might want to perform inference on scales which respect these bounds. For instance, one may prefer to treat $\log(\Theta_s)$ as the target estimand, with the assumption that $\Theta_s > 0$, or treat $\text{logit}(\Psi_s)$ as the target estimand, assuming $\Psi_s \in (0, 1)$. Here we sketch how one-step bias correction estimators could be constructed for these alternatives, and derive their asymptotic distributions.

First consider that, since the IC represents a pathwise derivatives for $\log(\Theta_s)$ and $\text{logit}(\Psi_s)$ respectively are

$$\frac{\phi_s(\boldsymbol{Z})}{\Theta_s}$$

$$\frac{\Phi_s(\boldsymbol{Z})}{\Psi_s(1 - \Psi_s)}$$

We let $\hat{\Theta}_s^0$ and $\hat{\Psi}_s^0$ be initial estimators such that $\hat{\Theta}_s^0 > 0$ and $\hat{\Psi}_s^0 \in (0,1)$. E.g.

$$\begin{aligned}
\hat{\Theta}_s^0 &= n^{-1}\sum_{i=1}^{n}\{\hat{\tau}(x_i) - \hat{\tau}_s(x_i)\}^2 \\
\hat{\Theta}_p^0 &= \hat{\Theta}_s^0 + n^{-1}\sum_{i=1}^{n}\left[\hat{\tau}_s(x_i) - \left\{n^{-1}\sum_{i=1}^{n}\hat{\tau}_s(x_i)\right\}\right]^2 \\
\hat{\Psi}_s^0 &= \frac{\hat{\Theta}_s^0}{\hat{\Theta}_p^0}
\end{aligned}$$

Using these initial estimators one can construct the one-step bias corrected estimators

$$\log(\hat{\Theta}_s^0) + \frac{n^{-1}\sum_{i=1}^{n}\hat{\phi}_s(\boldsymbol{z}_i)}{\hat{\Theta}_s^0}$$

$$\operatorname{logit}(\hat{\Psi}_s^0) + \frac{n^{-1}\sum_{i=1}^{n}\hat{\Phi}_s(\boldsymbol{z}_i)}{\hat{\Psi}_s^0(1-\hat{\Psi}_s^0)}$$

which we rewrite in terms of the estimators in the main text as

$$\log(\hat{\Theta}_s^0) + \frac{(\hat{\Theta}_s - \hat{\Theta}_s^0)}{\hat{\Theta}_s^0} \tag{16}$$

$$\operatorname{logit}(\hat{\Psi}_s^0) + \frac{\hat{\Theta}_p}{\hat{\Theta}_p^0}\frac{(\hat{\Psi}_s - \hat{\Psi}_s^0)}{\hat{\Psi}_s^0(1-\hat{\Psi}_s^0)} \tag{17}$$

where we have used the fact that

$$\begin{aligned}
n^{-1}\sum_{i=1}^{n}\hat{\Phi}_s(\boldsymbol{z}_i) &= \frac{n^{-1}\sum_{i=1}^{n}\hat{\phi}_s(\boldsymbol{z}_i) - \hat{\Psi}_s^0 n^{-1}\sum_{i=1}^{n}\hat{\phi}_p(\boldsymbol{z}_i)}{\hat{\Theta}_p^0} \\
&= \frac{(\hat{\Theta}_s - \hat{\Theta}_s^0) - \hat{\Psi}_s^0(\hat{\Theta}_p - \hat{\Theta}_p^0)}{\hat{\Theta}_p^0} \\
&= \frac{\hat{\Theta}_p}{\hat{\Theta}_p^0}(\hat{\Psi}_s - \hat{\Psi}_s^0)
\end{aligned}$$

We remark that, unlike the estimators $\hat{\Theta}_s$ and $\hat{\Psi}_s$, the estimators in (16) and (17) depend on the initial estimators $\hat{\Theta}_s^0$ and $\hat{\Psi}_s^0$ in a non-trivial way. We derive asymptotic distributions of these estimators given additional conditions on these initial estimators.

Theorem 3. Assume the conditions of Theorem 1 hold, $\Theta_s > 0$, and $(\Theta_s - \hat{\Theta}_s^0)/\hat{\Theta}_s^0 = o_P(n^{-1/4})$, then the estimator in (16) converges to $\log(\Theta_s)$ in probability, with a difference that, when multiplied by $n^{1/2}$, converges to a mean-zero normal random variable, with variance $||\phi_s(\boldsymbol{Z})||^2/\Theta_s^2$.

Proof. Under the conditions of Theorem 1, then $\hat{\Theta}_s$ is regular asymptotically linear, i.e. we can write

$$\hat{\Theta}_s = \Theta_s + n^{-1}\sum_{i=1}^{n}\phi_s(\boldsymbol{z}_i) + o_P(n^{-1/2})$$

Hence,

$$\log(\hat{\Theta}_s^0) + \frac{(\hat{\Theta}_s - \hat{\Theta}_s^0)}{\hat{\Theta}_s^0} - \log(\Theta_s) = n^{-1}\sum_{i=1}^{n}\frac{\phi_s(\boldsymbol{z}_i)}{\hat{\Theta}_s^0} + \frac{o_P(n^{-1/2})}{\hat{\Theta}_s^0} + \frac{\Theta_s - \hat{\Theta}_s^0}{\hat{\Theta}_s^0} - \log\left(1 + \frac{\Theta_s - \hat{\Theta}_s^0}{\hat{\Theta}_s^0}\right)$$

Using the Taylor series of $\log(1+x)$

$$\begin{aligned}
&= n^{-1}\sum_{i=1}^{n}\frac{\phi_s(\boldsymbol{z}_i)}{\hat{\Theta}_s^0} + \frac{o_P(n^{-1/2})}{\hat{\Theta}_s^0} + \sum_{j=2}^{\infty}\frac{(-1)^{j+1}}{j}\left(\frac{\Theta_s - \hat{\Theta}_s^0}{\hat{\Theta}_s^0}\right)^j \\
&= \left\{n^{-1}\sum_{i=1}^{n}\frac{\phi_s(\boldsymbol{z}_i)}{\Theta_s} + \frac{o_P(n^{-1/2})}{\Theta_s}\right\}(1+\hat{u}) + \sum_{j=2}^{\infty}\frac{(-1)^{j+1}\hat{u}^j}{j}
\end{aligned}$$

where $\hat{u} \equiv (\Theta_s - \hat{\Theta}_s^0)/\hat{\Theta}_s^0 = o_P(n^{-1/4})$ thus

$$\log(\hat{\Theta}_s^0) + \frac{(\hat{\Theta}_s - \hat{\Theta}_s^0)}{\hat{\Theta}_s^0} - \log(\Theta_s) = n^{-1}\sum_{i=1}^{n}\frac{\phi_s(\boldsymbol{z}_i)}{\Theta_s} + o_P(n^{-1/2})$$

□

Theorem 4. Assume the conditions of Theorem 2 hold, $\Psi_s \in (0,1)$, $(\Psi_s - \hat{\Psi}_s^0)/\{\hat{\Psi}_s^0(1 - \hat{\Psi}_s^0)\} = o_P(n^{-1/4})$, and $(\Theta_p - \hat{\Theta}_p^0)/\hat{\Theta}_p^0 = o_P(n^{-1/4})$ then the estimator in (17) converges to $\text{logit}(\Psi_s)$ in probability, with a difference that, when multiplied by $n^{1/2}$, converges to a mean-zero normal random variable, with variance $||\Phi_s(\boldsymbol{Z})||^2/\{\Psi_s(1-\Psi_s)\}^2$.

Proof. Under the conditions of Theorem 2, then $\hat{\Psi}_s$ is regular asymptotically linear, i.e. we can write

$$\hat{\Psi}_s = \Psi_s + n^{-1}\sum_{i=1}^{n}\Phi_s(\boldsymbol{z}_i) + o_P(n^{-1/2})$$

Also, using the Taylor series of $\log(1 \pm x)$ note that for arbitrary values a, b

$$\begin{aligned}
\text{logit}(a) - \text{logit}(b) &= \log\left(1 + \frac{a-b}{b}\right) - \log\left(1 - \frac{a-b}{1-b}\right) \\
&= \sum_{j=1}^{\infty} \frac{(-1)^{j+1}}{j}\left(\frac{a-b}{b}\right)^j + \sum_{j=1}^{\infty} \frac{1}{j}\left(\frac{a-b}{1-b}\right)^j \\
&= \sum_{j=1}^{\infty} \left(\frac{a-b}{b(1-b)}\right)^j \frac{(b^j - (b-1)^j)}{j}
\end{aligned}$$

Hence,

$$\begin{aligned}
&\text{logit}(\hat{\Psi}_s^0) + \frac{\hat{\Theta}_p}{\hat{\Theta}_p^0}\frac{(\hat{\Psi}_s - \hat{\Psi}_s^0)}{\hat{\Psi}_s^0(1-\hat{\Psi}_s^0)} - \text{logit}(\Psi_s) \\
&= \left[n^{-1}\sum_{i=1}^{n}\frac{\Phi_s(\boldsymbol{z}_i)}{\hat{\Psi}_s^0(1-\hat{\Psi}_s^0)} + \frac{(\Psi_s - \hat{\Psi}_s^0)}{\hat{\Psi}_s^0(1-\hat{\Psi}_s^0)} + \frac{o_P(n^{-1/2})}{\hat{\Psi}_s^0(1-\hat{\Psi}_s^0)}\right]\frac{\hat{\Theta}_p}{\hat{\Theta}_p^0} \\
&- \sum_{j=1}^{\infty}\left(\frac{\Psi_s - \hat{\Psi}_s^0}{\hat{\Psi}_s^0(1-\hat{\Psi}_s^0)}\right)^j \frac{((\hat{\Psi}_s^0)^j - (\hat{\Psi}_s^0 - 1)^j)}{j} \\
&= \left[n^{-1}\sum_{i=1}^{n}\frac{\Phi_s(\boldsymbol{z}_i)}{\Psi_s(1-\Psi_s)} + \frac{o_P(n^{-1/2})}{\Psi_s(1-\Psi_s)}\right]\frac{\hat{\Theta}_p}{\hat{\Theta}_p^0}\left(1 + \frac{\Psi_s(1-\Psi_s) - \hat{\Psi}_s^0(1-\hat{\Psi}_s^0)}{\hat{\Psi}_s^0(1-\hat{\Psi}_s^0)}\right) \\
&+ \hat{v}\hat{u} - \sum_{j=2}^{\infty}\hat{u}^j\frac{((\hat{\Psi}_s^0)^j - (\hat{\Psi}_s^0 - 1)^j)}{j}
\end{aligned}$$

where $\hat{u} \equiv (\Psi_s - \hat{\Psi}_s^0)/\{\hat{\Psi}_s^0(1-\hat{\Psi}_s^0)\} = o_P(n^{-1/4})$ and

$$\begin{aligned}
\hat{v} &= \frac{\hat{\Theta}_p}{\hat{\Theta}_p^0} - 1 \\
&= \frac{\hat{\Theta}_p - \Theta_p}{\hat{\Theta}_p^0} + \frac{\Theta_p - \hat{\Theta}_p^0}{\hat{\Theta}_p^0} = o_p(n^{-1/4})
\end{aligned}$$

where the last line follows since $\hat{\Theta}_p - \Theta_p = o_p(n^{-1/4})$. Also

$$\frac{\hat{\Theta}_p}{\hat{\Theta}_p^0}\left\{1 + \frac{\Psi_s(1-\Psi_s) - \hat{\Psi}_s^0(1-\hat{\Psi}_s^0)}{\hat{\Psi}_s^0(1-\hat{\Psi}_s^0)}\right\} = (1+\hat{v})\left\{1 + \hat{u}(1 - \Psi_s - \hat{\Psi}_s^0)\right\} \xrightarrow{p} 1$$

Therefore we recover

$$\text{logit}(\hat{\Psi}_s^0) + \frac{\hat{\Theta}_p}{\hat{\Theta}_p^0} \frac{(\hat{\Psi}_s - \hat{\Psi}_s^0)}{\hat{\Psi}_s^0(1 - \hat{\Psi}_s^0)} - \text{logit}(\Psi_s) = n^{-1} \sum_{i=1}^{n} \frac{\Phi_s(\boldsymbol{z}_i)}{\Psi_s(1 - \Psi_s)} + o_P(n^{-1/2}).$$

□

D.6 Defining Treatment effects on different scales

In the current work, we examine the importance of variable subsets in predicting the causal contrast $Y^1 - Y^0$, and hence the CATE $\tau(\boldsymbol{x})$. Our conclusions regarding heterogeneity depend on this choice of scale, and different conclusions could be reached if one had considered another effect definition. For instance, supposing that $Y > 0$ almost surely, then one might be interested in VIMs with respect to the conditional risk ratio

$$\psi(\boldsymbol{x}) \equiv \log\{E(Y^1|\boldsymbol{X} = \boldsymbol{x})\} - \log\{E(Y^0|\boldsymbol{X} = \boldsymbol{x})\}. \tag{18}$$

It is possible that $\psi(\boldsymbol{x})$ is constant (suggesting no heterogeneity), but $\tau(\boldsymbol{x})$ is not constant (suggesting some heterogeneity), or vice-versa. Similar problems apply to conditional odds ratios, where for a binary outcome $Y \in \{0, 1\}$, one replaces the logarithms in (18), with the logit function. It is an open topic of debate, how treatment effects should be communicated to clinicians in such settings We recommend that practitioners remain aware of scale dependencies when using TE-VIMs.

D.7 Linear projections of the CATE

The ideas in the current paper have been extended in the direction of nonparametric linear projection parameters by Boileau et al. (2023) . They propose using the estimands

$$\beta_j \equiv \arg\min_{\beta \in \mathbb{R}} L\{\tau_p + \beta[X_j - E(X_j)]\} = \frac{\text{cov}(Y^1 - Y^0, X_j)}{\text{var}(X_j)}$$

as a proxy for the importance of a covariate X_j. Since these estimands are not invariant to the scale on which X_j is defined, hence the authors determine relative variable importance based on the null hypothesis tests that each $\beta_j = 0$. These estimands are generally less sensitive to non-linearities and parameter interactions than TE-VIMs. For instance, it may be the case that X_j is important in explaining treatment effect heterogeneity, but $\beta_j = 0$ in truth. Inference of β_j has no power to detect such covariates. That said, the linear term β_j remains scientifically interesting, e.g. in roughly determining covariate thresholds for further investigation.

D.8 Treatment effect cumulative distribution function

A related proposal considers the treatment effect cumulative distribution function (TE-CDF) (Levy and van der Laan, 2018) , which is a curve $\beta : \mathbb{R} \mapsto [0,1]$, with $\beta(t) = Pr\{\tau(\boldsymbol{X}) \leq t\}$.

Motivated by OTRs, the value $\beta(0)$ is of particular interest since it captures the marginal probability that an individual has a negative CATE, and therefore the proportion of the population which is not treated under the OTR. We note that $\beta(0)$ is not the same as $Pr(Y^1 - Y^0 \leq 0)$ which suffers similar identifiability issues regarding the joint distribution of (Y^1, Y^0) as the quantity $\text{var}(Y^1 - Y^0)$ mentioned previously. Like the OTR-VIMs above, the TE-CDF is generally not pathwise differentiable, hence Levy and van der Laan (2018) focus instead on a kernel smoothed analogue of $\beta(t)$. It is mentioned by Levy et al. (2021) that, provided $\tau_p > 0$, then Chebyshev's inequality implies $\beta(0) \leq \Lambda \equiv \Theta_p/\tau_p^2$.

Thus, the VTE is also of scientific interest since it can be used to bound $\beta(0)$, informing investigators about the probability of negative CATEs once a positive ATE has been established. Estimation of Λ could be carried out using estimating equations estimators, as in the current work, or targeted methods (Levy et al., 2021), using the IC for Λ,

$$\frac{\{\varphi(\boldsymbol{Z}) - \tau_p\}^2 - \{\varphi(\boldsymbol{Z}) - \tau(\boldsymbol{X})\}^2 - \Lambda\tau_p\{2\varphi(\boldsymbol{Z}) - \tau_p\}}{\tau_p^2}.$$

Below we briefly sketch the details for the estimating equations estimator.

First note Chebyshev's inequality: For a variable V with mean μ and variance σ^2, for $k > 0$

$$\begin{aligned} Pr(|V - \mu| \geq k\sigma) &\leq k^{-2} \\ Pr(V \geq \mu + k\sigma) + Pr(V \leq \mu - k\sigma) &\leq k^{-2} \end{aligned}$$

which implies the weaker inequality,

$$Pr(V \leq \mu - k\sigma) \leq k^{-2}$$

Let $\tau(\boldsymbol{X})$ be the CATE with ATE τ_p and VTE Θ_p then,

$$\beta(0) = Pr\{\tau(\boldsymbol{X}) \leq 0\} = Pr\left\{\tau(\boldsymbol{X}) \leq \tau_p - \left(\frac{\tau_p}{\sqrt{\Theta_p}}\right)\sqrt{\Theta_p}\right\} \leq \frac{\Theta_p}{\tau_p^2}$$

Where the inequality applies only when $\tau_p > 0$. It follows that, when the ATE is positive, the quantity on the RHS bounds $\beta(0)$ from above. The quotient rule gives that the IC

(pathwise derivative) is,

$$\begin{aligned}\phi_\beta(\boldsymbol{Z}) &= \frac{1}{\tau_p^2}\phi_p(\boldsymbol{Z}) - 2\left(\frac{\Theta_p}{\tau_p^3}\right)\{\varphi(\boldsymbol{Z}) - \tau_p\} \\ &= \frac{\{\varphi(\boldsymbol{Z}) - \tau_p\}^2 - \{\varphi(\boldsymbol{Z}) - \tau(\boldsymbol{X})\}^2 - \left(\frac{\Theta_p}{\tau_p^2}\right)\tau_p\{2\varphi(\boldsymbol{Z}) - \tau_p\}}{\tau_p^2}\end{aligned}$$

where $\{\varphi(\boldsymbol{Z}) - \tau_p\}$ is the IC of τ_p. An estimating equations estimator is that which solves

$$n^{-1}\sum_{i=1}^{n}\hat{\phi}_\beta(\boldsymbol{z}_i) = 0$$

where $\hat{\phi}_\beta(\boldsymbol{z})$ is an estimate of $\phi_\beta(\boldsymbol{z})$. Therefore $\hat{\Theta}_p/\hat{\tau}_p^2$ is an estimating equations estimator where $\hat{\Theta}_p$ is the VTE estimator in the current paper and $\hat{\tau}_p$ is the AIPW estimator of the ATE.

D.9 Continuous analogue estimands

Let $\lambda(\boldsymbol{x}) \equiv \mathrm{cov}(A, Y|\boldsymbol{X} = \boldsymbol{x})/\mathrm{var}(A|\boldsymbol{X} = \boldsymbol{x})$. Consider the loss $L_{P_0}\{f\} \equiv ||\lambda(\boldsymbol{x}) - f(\boldsymbol{x})||^2$. Applying the same approach as in Appendix A, we see that this loss has IC

$$\{\lambda(\tilde{\boldsymbol{x}}) - f(\tilde{\boldsymbol{x}})\}^2 - L_{P_0}\{f\} + 2E[\{\lambda(\boldsymbol{x}) - f(\boldsymbol{x})\}\partial_t\lambda_t(\boldsymbol{x})] \tag{19}$$

where $\lambda_t(\boldsymbol{x})$ denotes $\lambda(\boldsymbol{x})$ evaluated under P_t. We will show that,

$$\partial_t\lambda(\boldsymbol{x}) = \frac{\tilde{f}(\boldsymbol{x})}{f(\boldsymbol{x})}\{\tilde{y} - \mu(\boldsymbol{x}) - \lambda(\boldsymbol{x})\{\tilde{a} - \pi(\boldsymbol{x})\}\}\frac{\tilde{a} - \pi(\boldsymbol{x})}{\mathrm{var}(A|\boldsymbol{X} = \boldsymbol{x})} \tag{20}$$

and hence, letting

$$\varphi_\lambda(\boldsymbol{z}) \equiv \{y - \mu(\boldsymbol{x}) - \lambda(\boldsymbol{x})\{a - \pi(\boldsymbol{x})\}\}\frac{a - \pi(\boldsymbol{x})}{\mathrm{var}(A|\boldsymbol{X} = \boldsymbol{x})} + \lambda(\boldsymbol{x})$$

then the IC of $L_{P_0}\{f\}$ is

$$\{\varphi_\lambda(\boldsymbol{z}) - f(\tilde{\boldsymbol{x}})\}^2 - \{\varphi_\lambda(\boldsymbol{z}) - \lambda(\tilde{\boldsymbol{x}})\}^2 - L_{P_0}\{f\}. \tag{21}$$

Just as with the $L^*\{f\}$ loss in the main paper, this implies the IC of $E\left[\mathrm{var}\{\lambda(\boldsymbol{X})|\boldsymbol{X}_{-s}\}\right]$ is,

$$\{\varphi_\lambda(\boldsymbol{z}) - \lambda_s(\boldsymbol{x})\} - \{\varphi_\lambda(\boldsymbol{z}) - \lambda(\boldsymbol{x})\} - E\left[\mathrm{var}\{\lambda(\boldsymbol{X})|\boldsymbol{X}_{-s}\}\right]$$

where $\lambda_s(\boldsymbol{x}) = E\{\lambda(\boldsymbol{X})|\boldsymbol{X}_{-s} = \boldsymbol{x}_{-s}\}$. The IC for $\text{var}\{\lambda(\boldsymbol{X})\}$ follows as a special case where s includes all the observed covariates. Additionally, by (5) the IC of $E\{\lambda(\boldsymbol{X})\}$ is,

$$\varphi_\lambda(\boldsymbol{z}) - E\{\lambda(\boldsymbol{X})\}.$$

To demonstrate (20) we first note that, by (4),

$$\begin{aligned}\partial_t \text{cov}_{P_t}(A, Y|\boldsymbol{X} = \boldsymbol{x}) &= \partial_t E_{P_t}\{[A - E_{P_t}(A|\boldsymbol{X})][Y - E_{P_t}(Y|\boldsymbol{X})]|\boldsymbol{X} = \boldsymbol{x}\} \\ &= \frac{\tilde{f}(\boldsymbol{x})}{f(\boldsymbol{x})}\left[\{\tilde{a} - \pi(\boldsymbol{x})\}\{\tilde{y} - \mu(\boldsymbol{x})\} - \text{cov}_P(A, Y|\boldsymbol{X} = \boldsymbol{x})\right]\end{aligned}$$

We also obtain $\partial_t \text{var}_{P_t}(A|\boldsymbol{X} = \boldsymbol{x})$ as a special case of the above expression when $Y = A$. By the quotient rule,

$$\begin{aligned}\partial_t \frac{\text{cov}_{P_t}(A, Y|\boldsymbol{X} = \boldsymbol{x})}{\text{var}_{P_t}(A|\boldsymbol{X} = \boldsymbol{x})} &= \frac{\partial_t \text{cov}_{P_t}(A, Y|\boldsymbol{X} = \boldsymbol{x})}{\text{var}_P(A|\boldsymbol{X} = \boldsymbol{x})} - \frac{\text{cov}_P(A, Y|\boldsymbol{X} = \boldsymbol{x})}{\text{var}_P(A|\boldsymbol{X} = \boldsymbol{x})} \frac{\partial_t \text{var}_{P_t}(A, Y|\boldsymbol{X} = \boldsymbol{x})}{\text{var}_P(A|\boldsymbol{X} = \boldsymbol{x})} \\ &= \frac{\tilde{f}(\boldsymbol{x})}{f(\boldsymbol{x})}\{\tilde{y} - \mu(\boldsymbol{x}) - \lambda(x)\{\tilde{a} - \pi(\boldsymbol{x})\}\}\frac{\tilde{a} - \pi(\boldsymbol{x})}{\text{var}(A|\boldsymbol{X} = \boldsymbol{x})}\end{aligned}$$

Thus, the result follow.

E Issues with the S-learner of the CATE

A simple alternative to the T-learner is the S-learner (Künzel et al., 2019), with both being based on the decomposition $\tau(\boldsymbol{x}) = \mu(1, \boldsymbol{x}) - \mu(0, \boldsymbol{x})$. The S-learner estimate of the CATE is $\hat{\tau}^{(S)}(\boldsymbol{x}) \equiv \hat{\mu}^*(1, \boldsymbol{x}) - \hat{\mu}^*(0, \boldsymbol{x})$, where $\hat{\mu}^*(a, \boldsymbol{x})$ represents an estimate of $\mu(a, \boldsymbol{x})$ obtained by a regression of Y on (A, X) using all of the data. This is similar to the T-learner, except the S-learner uses a 'single' regression $\hat{\mu}^*(a, \boldsymbol{x})$, and the T-learner uses 'two' regressions to estimate $\hat{\mu}(1, \boldsymbol{x})$ and $\hat{\mu}(0, \boldsymbol{x})$.

The main issue with the S-learner vs. the T-learner is that $\hat{\mu}^*(a, \boldsymbol{x})$ is chosen to make an optimal bias-variance trade-off over the population distribution of treatment and covariates. When there is poor overlap between the treated and untreated subgroups (e.g. treatment is correlated with covariates), then regularization biases, which control this trade-off, may bias the effect of treatment on outcome towards zero, making the S-learner potentially poorly targeted towards CATE estimation.

In practice, this means that the S-learner is more likely to produce extremely small or even negative VTE estimates. For instance, if we replace $\hat{\mu}(a, \boldsymbol{x})$ with $\hat{\mu}^*(a, \boldsymbol{x})$ in all algorithms, then the results for our applied example are severely affected. In particular, Figures 9 and 10 show the resulting unscaled and scaled TE-VIMs. We see that Algorithms

based on the (modified) DR-learner (noSS-B and SS-B) are less affected when compared with the corresponding figures in the main paper, but Algorithms based on the S-learner (noSS-A and noSS-A) give wildly different results.

AIPW estimates of the ATE using the pseudo outcomes from the modified Algorithms noSS, SS-A, and SS-B were similar, respectively: $29.6mm^{-3}$ (CI: 15.5, 43.8; p<0.01); $28.8mm^{-3}$ (CI: 14.1, 43.5; p<0.01); $28.9mm^{-3}$ (CI: 14.2, 43.7; p<0.01). VTE estimates differed substantially between S- and (modified) DR-learner based algorithms. With Algorithms noSS-A and SS-A returning negative point estimates: $-3.80 \times 10^{-9} mm^{-6}$ (CI: -1.75×10^{-9}, 1.75×10^{-9}) and $-104mm^{-6}$ (CI: -175, -33.0), while Algorithms noSS-B and SS-B giving positive estimates: $2700mm^{-6}$ (CI: 1300, 4090) and $1700mm^{-6}$ (CI: -610, 4020). For Algorithms SS-A and SS-B we obtain the square root of these VTE estimates are 51.9 and $41.3mm^{-3}$ respectively. Negative VTE estimates indicate that the S-learner of the CATE in Algorithms noSS-A and SS-A is a worse predictor of the pseudo-outcome than the sample mean pseudo-outcome (i.e. the AIPW estimate of the ATE based on $\hat{\mu}^*(a, \boldsymbol{x})$ and $\hat{\pi}(\boldsymbol{x})$).

F Applied example with Generalized Additive Models (GAMs)

The applied analysis example using ACTG175 data was rerun with the ‘SuperLearner‘ regression steps replaced with Generalized Additive Model regression, as implemented via the ‘gam‘ package in R Hastie (2004). Figures 9 and 10 show the resulting unscaled and scaled TE-VIMs. AIPW estimates of the ATE using the pseudo outcomes from the modified Algorithms noSS, SS-A, and SS-B were similar, respectively: $28.6mm^{-3}$ (CI: 14.3, 43.0; p<0.01); $28.8mm^{-3}$ (CI: 14.1, 43.4; p<0.01); $28.3mm^{-3}$ (CI: 13.7, 43.0; p<0.01). VTE estimates from Algorithms noSS-A, noSS-B, SS-A, and SS-B were respectively: $2665mm^{-6}$ (CI: 1059, 4271; p<0.01), $2759mm^{-6}$ (CI: 1114, 4405; p<0.01), $1147mm^{-6}$ (CI: -461, 2756; p= 0.16), and $1255mm^{-6}$ (CI: -468, 2977; p= 0.15).

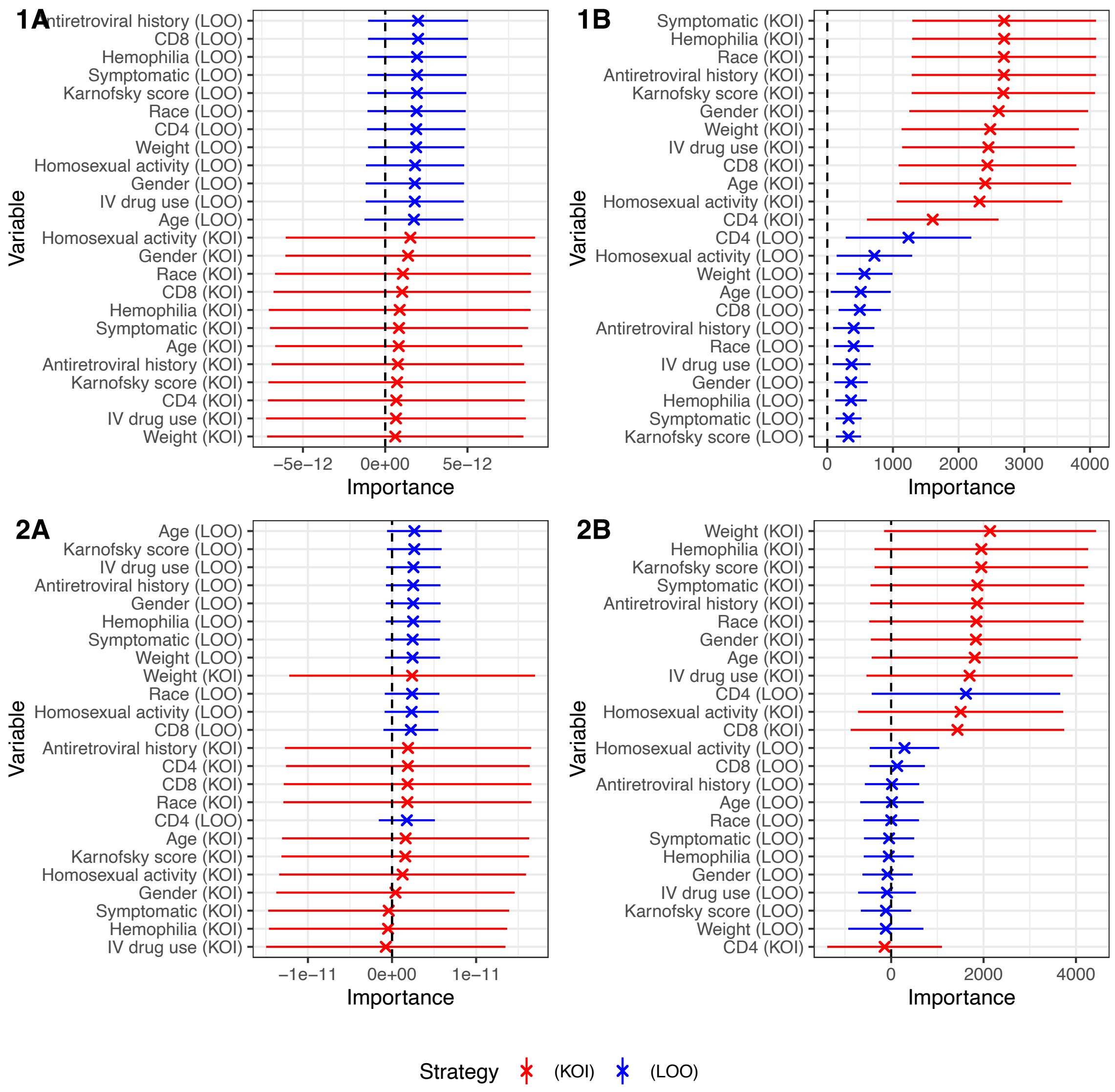

Figure 9: TE-VIM estimates $\hat{\Theta}_s$ from the ACTG175 study using modified Algorithms. Error bars indicate 95% CIs. In each plot, covariates are sorted according to their TE-VIM point estimate. Dashed lines indicate no importance. For the KOI mode, the TE-VIM represents the importance of the complement variable set, i.e. low-values denote high-importance of the KOI covariate.

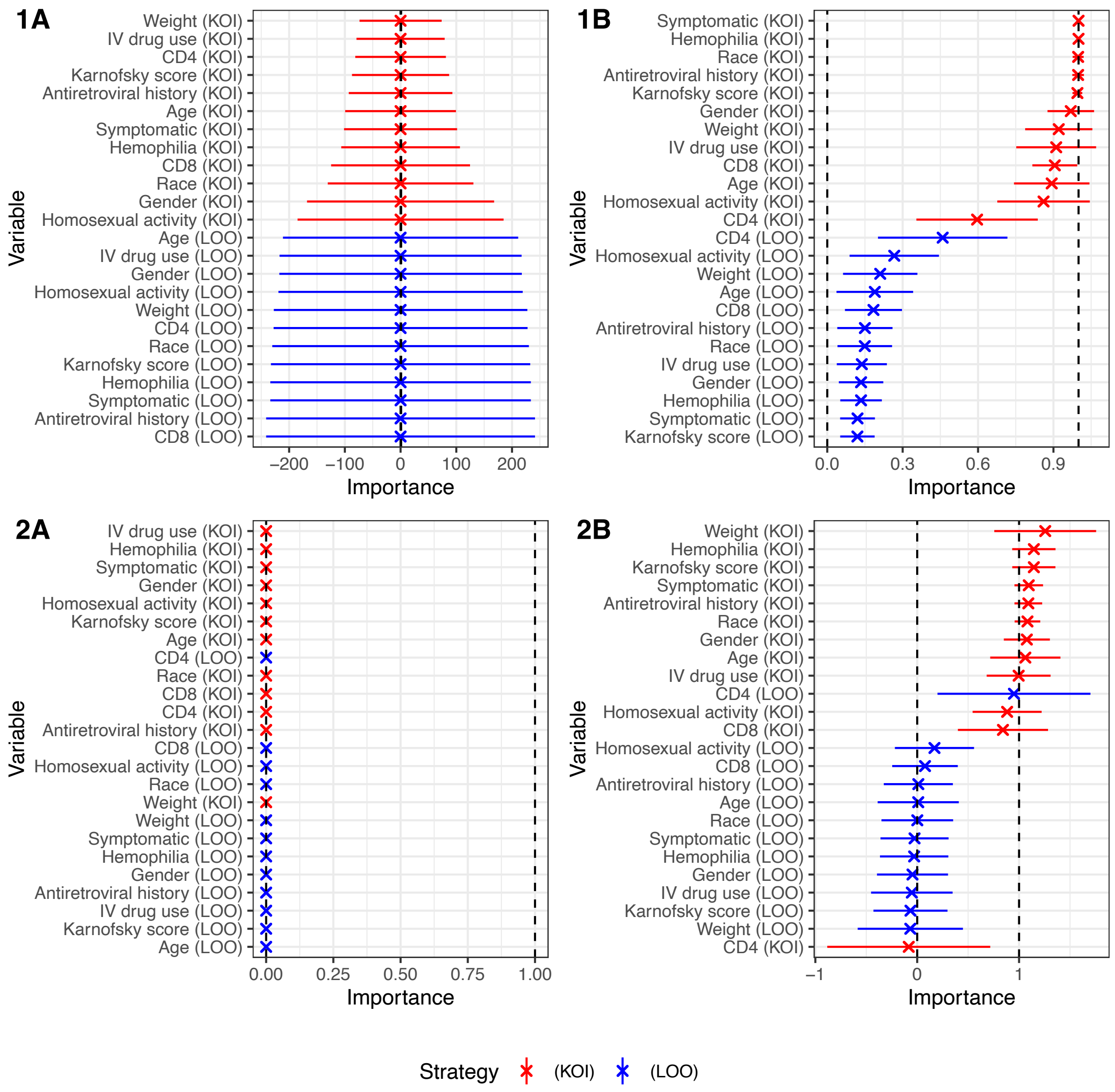

Figure 10: Scaled TE-VIM estimates $\hat{\Psi}_s$ from the ACTG175 study using modified Algorithms. Error bars indicate 95% CIs. In each plot, covariates are sorted according to their TE-VIM point estimate. Dashed lines indicate the $[0, 1]$ support of the scaled TE-VIM. For the KOI mode, the TE-VIM represents the importance of the complement variable set, i.e. low-values denote high-importance of the KOI covariate.

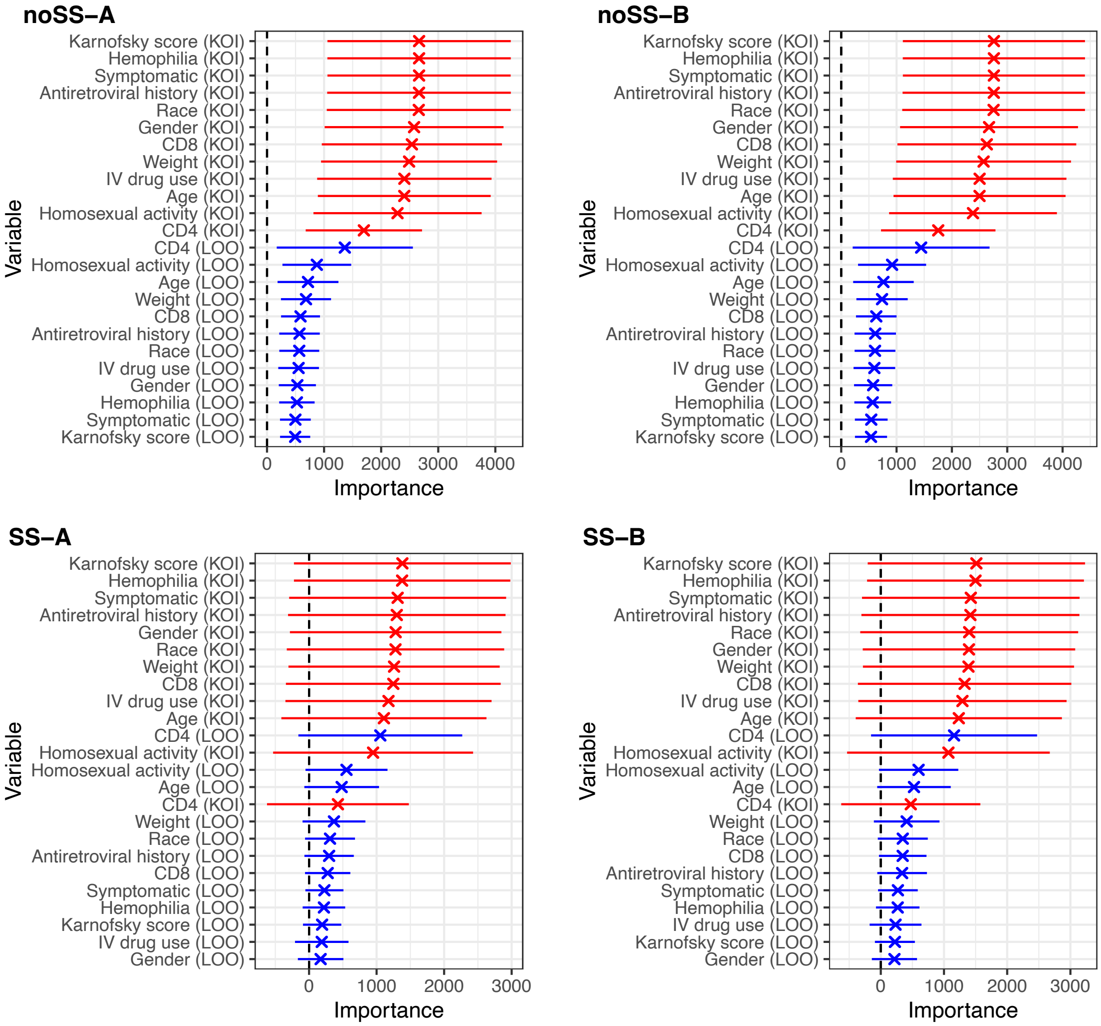

Figure 11: TE-VIM estimates $\hat{\Theta}_s$ from the ACTG175 study using modified Algorithms. Error bars indicate 95% CIs. In each plot, covariates are sorted according to their TE-VIM point estimate. Dashed lines indicate no importance. For the KOI mode, the TE-VIM represents the importance of the complement variable set, i.e. low-values denote high-importance of the KOI covariate.

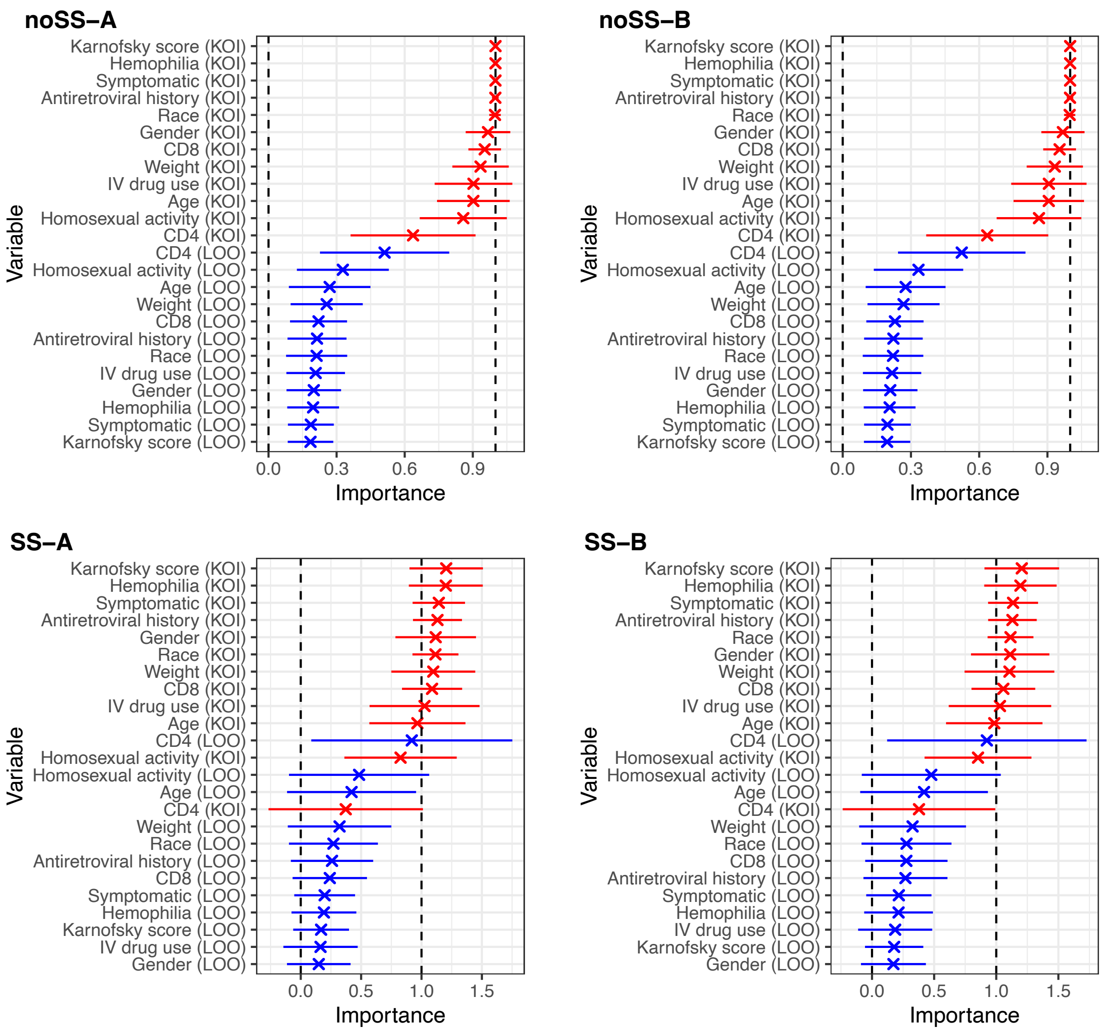

Figure 12: Scaled TE-VIM estimates $\hat{\Psi}_s$ from the ACTG175 study using modified Algorithms. Error bars indicate 95% CIs. In each plot, covariates are sorted according to their TE-VIM point estimate. Dashed lines indicate the $[0, 1]$ support of the scaled TE-VIM. For the KOI mode, the TE-VIM represents the importance of the complement variable set, i.e. low-values denote high-importance of the KOI covariate.
